# Different pathways of molecular pathophysiology underlie cognitive and motor tauopathy phenotypes in transgenic models for Alzheimer’s disease and frontotemporal lobar degeneration

**DOI:** 10.1007/s00018-014-1804-z

**Published:** 2014-12-19

**Authors:** V. Melis, C. Zabke, K. Stamer, M. Magbagbeolu, K. Schwab, P. Marschall, R. W. Veh, S. Bachmann, S. Deiana, P.-H. Moreau, K. Davidson, K. A. Harrington, J. E. Rickard, D. Horsley, R. Garman, M. Mazurkiewicz, G. Niewiadomska, C. M. Wischik, C. R. Harrington, G. Riedel, F. Theuring

**Affiliations:** 1 School of Medicine and Dentistry, University of Aberdeen, Aberdeen, Scotland, UK; 2 CCR/Institut für Pharmakologie, Charité-Universitätsmedizin Berlin, Institute of Pharmacology, Hessische Str. 3-4, 10115 Berlin, Germany; 3 Charité-Universitätsmedizin Berlin, Institute of Anatomy, Berlin, Germany; 4 Consultants in Veterinary Pathology Inc., Murrysville, PA USA; 5 Nencki Institute, Warsaw, Poland; 6 School of Medical Sciences, University of Aberdeen, Foresterhill, AB25 2ZD Scotland, UK

**Keywords:** Tau protein, Transgenic mice, P301L, Truncated cDNA Tau296-390, Motor impairment, Spatial cognition

## Abstract

A poorly understood feature of the tauopathies is their very different clinical presentations. The frontotemporal lobar degeneration (FTLD) spectrum is dominated by motor and emotional/psychiatric abnormalities, whereas cognitive and memory deficits are prominent in the early stages of Alzheimer’s disease (AD). We report two novel mouse models overexpressing different human tau protein constructs. One is a full-length tau carrying a double mutation [P301S/G335D; line 66 (L66)] and the second is a truncated 3-repeat tau fragment which constitutes the bulk of the PHF core in AD corresponding to residues 296–390 fused with a signal sequence targeting it to the endoplasmic reticulum membrane (line 1; L1). L66 has abundant tau pathology widely distributed throughout the brain, with particularly high counts of affected neurons in hippocampus and entorhinal cortex. The pathology is neuroanatomically static and declines with age. Behaviourally, the model is devoid of a higher cognitive phenotype but presents with sensorimotor impairments and motor learning phenotypes. L1 displays a much weaker histopathological phenotype, but shows evidence of neuroanatomical spread and amplification with age that resembles the Braak staging of AD. Behaviourally, the model has minimal motor deficits but shows severe cognitive impairments affecting particularly the rodent equivalent of episodic memory which progresses with advancing age. In both models, tau aggregation can be dissociated from abnormal phosphorylation. The two models make possible the demonstration of two distinct but nevertheless convergent pathways of tau molecular pathogenesis. L1 appears to be useful for modelling the cognitive impairment of AD, whereas L66 appears to be more useful for modelling the motor features of the FTLD spectrum. Differences in clinical presentation of AD-like and FTLD syndromes are therefore likely to be inherent to the respective underlying tauopathy, and are not dependent on presence or absence of concomitant APP pathology.

## Introduction

Neurodegeneration associated with abnormal processing of tau protein is increasingly recognised as having an important role in a number of societally important neurodegenerative disorders. Alzheimer’s disease was the first and most prevalent to be recognised as implicating the pathological aggregation of tau protein [1]. It is characterised histologically by the formation of intracellular neurofibrillary tangles (NFTs) first described by Alzheimer [2]. At the ultrastructural level the tangle is a dense array of Paired Helical Filaments (PHFs; [3]). These are de novo pathological polymers of which the principal constituent is a truncated, 100-amino acid fragment of the microtubule-associated protein tau [1, 4, 5]. This truncated fragment can catalyse the conversion of normal soluble tau into aggregated forms [6] which, in turn, can spread to neighbouring neurons [7–12]. The pattern of spread of tau-aggregation pathology in the human brain is highly stereotyped, and forms the basis of the six-step Braak staging system for neurofibrillary degeneration in AD [13]. A correlation between Braak stage and cognitive decline has been confirmed in numerous studies [14–18] with clinical dementia appearing at about stage 4 [18]. Stage 1 pathology appears already by the 4th–5th decade of life, preceding by some 27 years the appearance of amyloid β pathology [16, 19].

A poorly understood feature of tau-linked neurodegeneration is the very different clinical presentations of the associated syndromes. Whether or not linked to mutations in the MAPT gene, the clinical features of the frontotemporal lobar degeneration (FTLD) spectrum are generally dominated by motor and emotional/psychiatric abnormalities, whilst cognitive deficits tend to be rarer, more subtle and present at later disease stages [20]. In AD, on the other hand, the clinical features are dominated by cognitive and memory deficits occurring early in the disease, in the almost complete absence of motor abnormalities until very advanced disease stages. One possible explanation is that the difference depends on the presence of concomitant pathology linked to the amyloid β protein pathway. Indeed, animal studies of combined amyloid precursor protein (APP)/tau models have tended to emphasise tau as merely an aggravating or accelerating factor in what is conceptualised primarily as a disorder of APP processing [21]. However, given that 19 trials at phase 2 or 3 with drugs targeting various aspects of this pathway have so far failed to deliver convincing cognitive benefit [22] and that amyloid load is poorly correlated with cognitive impairment, it is not clear that an abnormality in APP processing contributes to cognitive impairment in AD. An alternative possibility is that the dissociation between motor and cognitive abnormalities seen in the various clinical tauopathies is actually inherent to differences in the underlying molecular pathophysiology of tau protein itself.

Familial autosomal dominantly inherited *MAPT* mutations have formed the basis of the majority of tau transgenic mouse models developed to date. The majority of these models express cDNA mutated in exon 10 (P301L, P301S, N279K), exon 9 (G272V), exon 13 (R406W) or exon 12 (V337M) and these present with intracellular aggregates of tau in neurons and glial cells (for review, see [23]). Motor phenotypes are common to most mutations in exon 10, while there is some suggestion that cognitive and emotional abnormalities may have a greater association with exons 9, 12 and 13 mutations [24]. We here report a novel FTDP-17 mouse model, in which the longest human *MAPT* cDNA in the central nervous system (htau40; 441 amino acids), containing 4 repeats and including point mutations P301S and G335D [termed Line 66 (L66)], was expressed under the Thy-1 regulatory element. Clinically, the P301S mutation causes early onset and rapid progression of disease associated with lowered microtubule assembly in patients and in mouse models with different tau isoforms (4R/0N [25] 4R/1N [26] 4R/2N [27]). The resulting L66 mice are characterised by severe neurofibrillary pathology associated with a prominent motor phenotype occurring in the absence of any higher cognitive features despite abundant tangles being present in hippocampus and entorhinal cortex. L66 mice also exhibit neuropathological features characteristic of a degenerative axonopathy.

Given the limitations of transgenic models based on MAPT mutations as models for AD, we have developed an alternative approach based on the truncated tau fragment restricted to the repeat domain which is the principal constituent of PHFs [28]. Cleavages at Glu-391 and/or Asp-421 result in tau fragments which appear relatively early in the disease state and induce toxicity in transfected cells in vitro [29, 30]. Rats transgenic for a truncated human tau fragment encompassing residues tau151–391 present with symptoms of neurodegeneration, intracellular accumulation of human tau in neurons [31], reduced life span [32], and late onset sensorimotor impairment (>9 months) [33]. We report here the development of a new tau transgenic line (Line 1, L1) in which mice express truncated tau296–390 similar to fragment of tau isolated from AD PHFs [1] and that is targeted to the endoplasmic reticulum (ER) membrane. We report here that L1 manifests a strong cognitive phenotype occurring in the absence of prominent sensorimotor features. The tau pathology seen in L1 remains at the stage of diffuse oligomeric aggregates, and does not progress histologically to tangles.

## Materials and methods

### Cloning of the constructs and generation of transgenic mice

L1 and L66 transgenic mice were generated using two different constructs. (a) Plasmid pSS296–390, which contains the human tau cDNA, encoding amino acids 296–390 of the longest human tau isoform (htau40) [34] was used to generate L1 mice. The plasmid contained an N-terminal signal sequence for insertion of the nascent polypeptide into the membrane of the ER, as well as 5′- and 3′-untranslated sequences targeting the mRNA to membrane-bound ribosomes and supporting insertion of the signal sequence into the ER membrane [35]. Neuronal expression was ensured through insertion of the construct into the murine Thy-1 expression cassette (pTSC21k) kindly provided by H. van der Putten, Basel [36]. (b) Plasmid pP301S/G335D containing the longest human isoform (htau40, 441amino acids) in which two point mutations at positions +900 (C → T) and +1,003 (G → A) were introduced by PCR-directed mutagenesis. These mutations alter the codons for the amino acid changes P301S and G335D, respectively. The cDNA was inserted into the Thy-1 cassette to generate L66 mice.

The vectors were cloned, sequences confirmed and a linearized *Not*I DNA fragment was microinjected into fertilized NMRI mouse eggs according to standard techniques [37]. Founder mice were identified by PCR using appropriate Thy-1 (5′-gcaggaggtgctcagggacagc-3′; 5′-taccagctggctgacctgtagc-3′) and Tau primers (5′-gagctccctcatccactaag-3′) resulting in 230 and 970 bp fragments for the L1 and L66 constructs, respectively. Genomic DNA was prepared using DNeasy™ tissue kit (Qiagen) and the number of integrations and copy number of transgenes were determined by Southern blot.

### Determination of copy numbers in different transgenic founders by Southern blot

DNA was prepared from tail biopsies, digested with *Eco*RI and separated electrophoretically on agarose gels. Restriction fragments were transferred onto a nylon membrane by capillary blotting and hybridised to *Nco*I/*Xba*I fragments of the Thy-1 promoter cassette. Wild-type alleles are recognised by an 8,200 bp *Eco*RI restriction fragment corresponding to two copies and transgenic allele is recognised by a approximately 5,000 bp *Eco*RI restriction fragment. Quantitative analysis was performed with ImageQuant^®^ software (Molecular Dynamics) with average intensity of all pixels in retrieved spots taken as measure.

### Animals

Founders were expanded with NMRI (Charles River, Germany) wild types before interbreeding heterozygous offspring for several generations to attain homozygous lines. Unless otherwise stated, all experiments were conducted using homozygous L1 and L66 mice with a preference for female gender in the behavioural characterisation. Mice were typically housed singly or in small colonies (up to 10) in Type 2 Macrolon or shoebox cages with free access to water and food in climatized holding rooms under a 12 h day night cycle (lights on at 7.00 a.m.). Experiments were carried out in accordance with the European Communities Council Directive (63/2010/EU), a project licence with local ethical approval under the UK Animals (Scientific Procedures) Act (1986) or in accordance with the German Law for Animal Protection (Tierschutzgesetz).

### Immunoblotting

Mouse brains were harvested from 4-month-old wild-type or L1, and 10- to 12-month-old wild-type or L66 animals. Brain (approximately 0.5 g) was homogenised in 1.5 ml sodium-phosphate (5 mM), sucrose (0.32 M, pH 7.0), MgCl_2_ (1 mM) and EGTA (1 mM) with protease inhibitors (Roche Complete PIC). Homogenates were diluted fivefold into gel sample buffer and samples (10 µl) separated by electrophoresis using 15 % SDS-PAGE gels. Truncated tau (297–391; dGAE; [4]) (50 ng) and full-length tau (T40; 250 ng) were included as positive antibody controls. Proteins were separated on gels using a Laemmli buffer system and blotted, using Tris (12.5 mM), glycine (96 mM) buffer (pH 8.8), onto PVDF membrane in a BioRad Mini Protean II gel electrophoresis tank. Blocking in buffer (5 % Marvel milk powder in PBS) lasted 30 min followed by incubation with primary antibodies and HRP-labelled secondary antibodies diluted in blocking buffer for 1 h and three 10-min washes between each antibody. Bound antibody was detected by enhanced chemiluminescence using Immun-Star HRP substrate (Bio-Rad) and a Carestream 2200PRO Imager. The following primary tau antibodies were used. (1) Monoclonal antibody (mAb) 7/51 recognises a tau epitope generic for all 6 tau CNS isoforms and an epitope that is located in the third tau repeat [38]. The epitope is occluded when tau is bound in a paired helical filament (PHF)-like immunochemical configuration but can be exposed by formic acid pre-treatment [39]. (2) N-terminal specific mAb 27/342 recognises a generic tau epitope between Ser-208 and Ser-238 [6]. (3) mAb 27/499 recognises a human-specific epitope between Gly-16 and Gln-26 [6]. (4) K9JA, a rabbit polyclonal antiserum raised against the C-terminal 243–441 residues of tau (Dako, A0024). (5) T46, a mAb specific to the C-terminal amino acids 404–441 of human tau (Invitrogen, 13-6400). All of these tau antibodies are phospho-independent. Secondary horseradish peroxidase conjugated antibodies were from Sigma (anti-rabbit) and Bio-Rad (anti-mouse). Primary antibodies were used as 1:10 dilutions of hybridoma supernatants (mAbs 7/51, 423 and 27/499), 1:1,000 for purified K9JA and T46 and 1:1,000 for secondary antibodies.

### Bielschowsky silver staining

Dewaxed sections were stained for 20 min at room temperature in darkness using 10 % silver nitrate or until coloured light brown, and washed three times with distilled water. They were then incubated in ammoniacal silver nitrate solution (20 %) for 20 min at room temperature, again washed three times, developed in ammoniacal silver nitrate including five drops of developer (20 ml of 37 % formaldehyde, 0.5 g citrate, 1 drop 65 % nitric acid, 100 ml distilled water) until gold-brown, washed again and dehydrated before mounting in Entellan (Merck).

### Primulin staining

The thioflavin S staining protocol described by Sun and co-workers [40] was adapted for use with primulin [41]. In brief, sections were dewaxed, rehydrated by passage through graded alcohols, antiquenched with 0.25 % KMnO_4_ and 1 % sodium borohydride and stained with 0.05 % primulin in 50 % ethanol. Photobleaching was blocked by concentrated phosphate buffer and sections were mounted with Vectashield.

### Quantification of tau-immunoreactive cells

Whole brains were removed from the skulls, fixed in 4 % paraformaldehyde in PBS (pH 7.4), and then wax embedded, sectioned, prepared and stained as described previously [42]. For explorative pathology, sections were taken from numerous parts of brain along the fronto-caudal axis. Particular interest centred on cortical structures and these were identified in repeat sections according to the stereotactic mouse atlas [43]; they included entorhinal cortex (ERC), hippocampus (Hip; polymorphic cells of dentate gyrus, CA3 and CA1), retrosplenial cortex (RSC), visual cortex (VC), auditory cortex (AC), subiculum (S), amygdala (A).

The primary antibody used for quantification was mAb 7/51 (1:350 dilution). Bound primary antibody was amplified using the Vectastain^®^ ABC Kit (Vector Laboratories) as per manufacturer and visualised using diaminobenzidine (DAB). Sections were counterstained with haematoxylin (Dr. K. Hollborn & Sons).

### Histological analysis of L66 mouse brains

For visualisation of disintegrative degeneration and qualitative assessment of tau pathology in brain of L66, mice were killed under terminal anaesthesia and then perfused with type 3 perfusion fix (EM Sciences), brain kept in skulls for 12–16 h in fixative before transferring to PBS storage buffer. Brains were embedded in MultiBrain^®^ blocks and free-floating cryosections (30 μm) made of the entire block. A set of every eighth section was stained by the same procedure, selected from: (a) amino cupric silver stain according to de Olmos et al. [44]; (b) immunostaining with biotinylated mAb AT8 (ThermoScientific; 100 µg/ml used at 1:5,000); (c) immunostained with antibody for glial fibrillary acidic protein (GFAP) (Dako; 1:20,000) followed by goat anti-rabbit Vecta Elite (Vector Laboratories) and (d) immunostained with mAb 7/51 (1:500) followed by horse anti-mouse Vecta Elite.

Qualitative staining with mAbs 7/51 and AT8 (pSer202/Thr205; [45]) was performed using EnVision double staining system (Dako). Polyclonal p-Tau (S-404) antibody (Santa Cruz) was used on sections at a dilution of 1:1,000.

### Electron microscopy

Spinal cord tissue from 8- to 9-month-old L66 mice (4 wild-type and 4 L66 mice in equal numbers of male and female genders) was removed from animals perfused through the abdominal aorta with 0.05 % glutaraldehyde and 3 % para-formaldehyde in 0.1 M sodium cacodylate buffer (pH 7.35) containing 4 % hydroxyethyl starch. Spinal cords were taken and immersed in the same fixative for 24 h at 4 °C, and cryoprotected in 2.3 M sucrose at 4 °C overnight. The tissue was then frozen in liquid nitrogen, and blocks of 1.0 mm^3^ sectioned at −80 °C. Ultrathin sections (70 nm) were retrieved at −100 °C in 2.3 M sucrose with 2 % methyl cellulose, and transferred to formvar/carbon-coated nickel grids. Immunolabeling entailed rinse of specimen in 0.1 M PBS followed by blocking for 30 min using 5 % goat serum (Sigma G9023) in 0.1 M PBS. Rabbit anti-tau (Sigma T6402; diluted 1:1,000 in 5 % goat serum/PBS) was applied at 4 °C overnight, washed repeatedly with goat serum/PBS and incubated with 10-nm gold-conjugated secondary antibody (Amersham RPN 421; diluted 1:40 in 5 % goat serum/PBS) at room temperature for 60 min. After washing with 0.1 M PBS, sections were fixed with glutaraldehyde (2.5 % in 0.06 M PBS) at room temperature for 5 min, rinsed with water, floated on a droplet of uranyl acetate (4 % in water) at 4 °C for 4 min and transferred to a droplet of 0.4 % uranyl acetate/2 % methyl cellulose. Labelled grids were dried for ≥30 min at room temperature and examined in a Zeiss EM906 electron microscope at 80 kV. The investigator was blinded with respect to the genotype.

### Behavioural phenotyping

#### Sensorimotor screen

A number of tests were conducted to determine sensory motor abilities, global gait function and motor learning in both L1 and L66 lines.

*Catwalk*: Gait analysis was performed on freely walking mice using the CatWalk (Noldus, Wageningen, The Netherlands). Three trials per mouse were administered enabling the mouse to traverse the glass plate from East to West (and vice versa) at a maximum of 5 s. Data were recorded automatically (Catwalk software version 7) including relative paw position (hind overlapping/non-overlapping with front paws), timing (stand, swing and step cycle), base of support, pressure and dimensions of each footfall, which enabled calculation of stride length and paw distance, regularity index (preferred paw placement sequence during walking). As for all behavioural tests, apparatus was wiped with 70 % ethanol between animals.

*Balance beam*: Sensorimotor coordination was tested using one set of square and one set of round balance beams (50-cm length; 28-, 12- or 5-mm in cross-section; 30° incline). Subjects were given one trial per beam (from big to small) and latency to traverse the beam was scored and averaged. Failure to traverse the beam during the allotted time terminated the trial and the maximum time (30 s) was recorded. Beams were wiped with 70 % ethanol between animals.

*RotaRod*: An automated 4-lane accelerating RotaRod (TSE, Germany) was used to examine motor coordination and motor learning. Testing consisted of four trials per day for 3 consecutive days with inter-trial intervals of 2–3 min. Mice were placed on a slowly rotating rod [1 rotation per minute (rpm)] and the rod was accelerated from 1 to 45 rpm over the trial time of 5 min. Trials were terminated when animals fell off the rod or the maximum time was achieved. Lanes were allocated following a Latin square design to counterbalance for both rod position and time of testing. The apparatus was cleaned with 70 % ethanol between trials.

#### Cognitive testing in open field water maze

The water maze was a cylindrical, grey Perspex pool (150-cm diameter; 50-cm height). It was filled with water (21 ± 1 °C) and a clear Perspex, circular, rigid escape platform (10-cm diameter, 35-cm height) was submerged about 1 cm below the water surface. It was placed in a laboratory with plenty extra-maze cues. The mice were released into the pool facing the wall at one of four cardinal points: North, East, South and West. Swim paths were registered with an overhead CCTV camera and online processed using the image analyser Ethovision 3.1Pro (Noldus IT, Wageningen, The Netherlands). All recordings were also stored on video tape or DVD.

*Reference memory*: The reference memory acquisition training was conducted over 4 days with six trials per day, and an inter-trial interval (ITI) of 10 min (Days 1–4). The platform was positioned within the centre of one quadrant of the pool and remained at the fixed location throughout. All four platform locations were used and counter balanced with genotype. A maximum trial length of 60 s was allowed with 30 s on the platform. Animals not finding the platform location during this time were gently guided to it. Data extracted from the recording included overall path length as spatial learning index, swim speed as a proxy for motor ability, and wall hugging (thigmotaxis) as an escape strategy implemented early in the learning phase and progressively diminishing.

One hour after the last day of acquisition training, a probe test was conducted. The platform was removed from the pool and mice were released facing the wall, directly opposite the training quadrant and were allowed to swim freely for 60 s. Time spent in each quadrant was used as an index of spatial bias.

*Problem solving*: The protocol was adapted from Chen and co-workers [46] and included a cued training phase followed by the training to criterion test involving a series of spatial learning tasks (“problems”) with a hidden platform. Cued training: the pool was surrounded by white curtains to occlude sight of extra-maze cues. The platform was cued by a flag placed onto it (approximately 15-cm high) and the platform location was changed for each trial in a predetermined fashion to one of 20 possible sites. Animals were trained for 4 consecutive days and received 4 trials per day. Training to criterion: the curtains were withdrawn to reveal extra maze cues and the platform submerged about 1 cm below the water surface. Each animal was trained for up to eight trials per day for a maximum of 2 weeks to a rigorous performance criterion of three successive trials with an escape latency of less than 20 s. If the criterion was achieved before the eight daily trials were given, then the session for this animal was terminated and a new problem (new platform location) was implemented on the following day. The number of trials required to reach criterion for each problem was calculated as the primary measure of the animal’s learning capacity.

#### Anxiety test: light/dark box

Line 1 mice (7–8 months) were tested in the light/dark box. The apparatus consisted of two Perspex boxes: an open white compartment (30 cm length × 30 cm wide × 30 cm height) brightly illuminated and a smaller dark compartment (30 cm length × 20 cm wide × 25 cm height) covered with a dark Perspex lid. The compartments were connected by an aperture at floor level. Animals were individually placed into the brightly lit compartment facing the opening and allowed to freely explore the apparatus for 10 min. Time spent and numbers of entries into each compartment were extracted from video-tracked recordings (Ethovision XT6) as indicators of anxiety.

### Data analysis

Results are presented as mean ± SE and were analysed using parametric statistics. Typically, comparisons applied factorial analysis of variance (ANOVA) with genotype as between-subject and trial/day/age/region as within-subject factor followed by Bonferroni corrected *t* tests, or used simple 1-way ANOVA followed post hoc by Student’s *t* test. Contrast with chance was confirmed by Student’s *t* test. All analyses were two tailed, conducted using Graph Pad Prism (V 4.01), and applied a 95 % confidence level (*p* < 0.05).

## Results

### Over-expression of mutant full-length tau in L66 is characterised by intracellular tau fibrils with a proteolytically stable repeat domain core, axonal disintegration, and a prominent motor phenotype

#### General observations

**Fig. 1:**
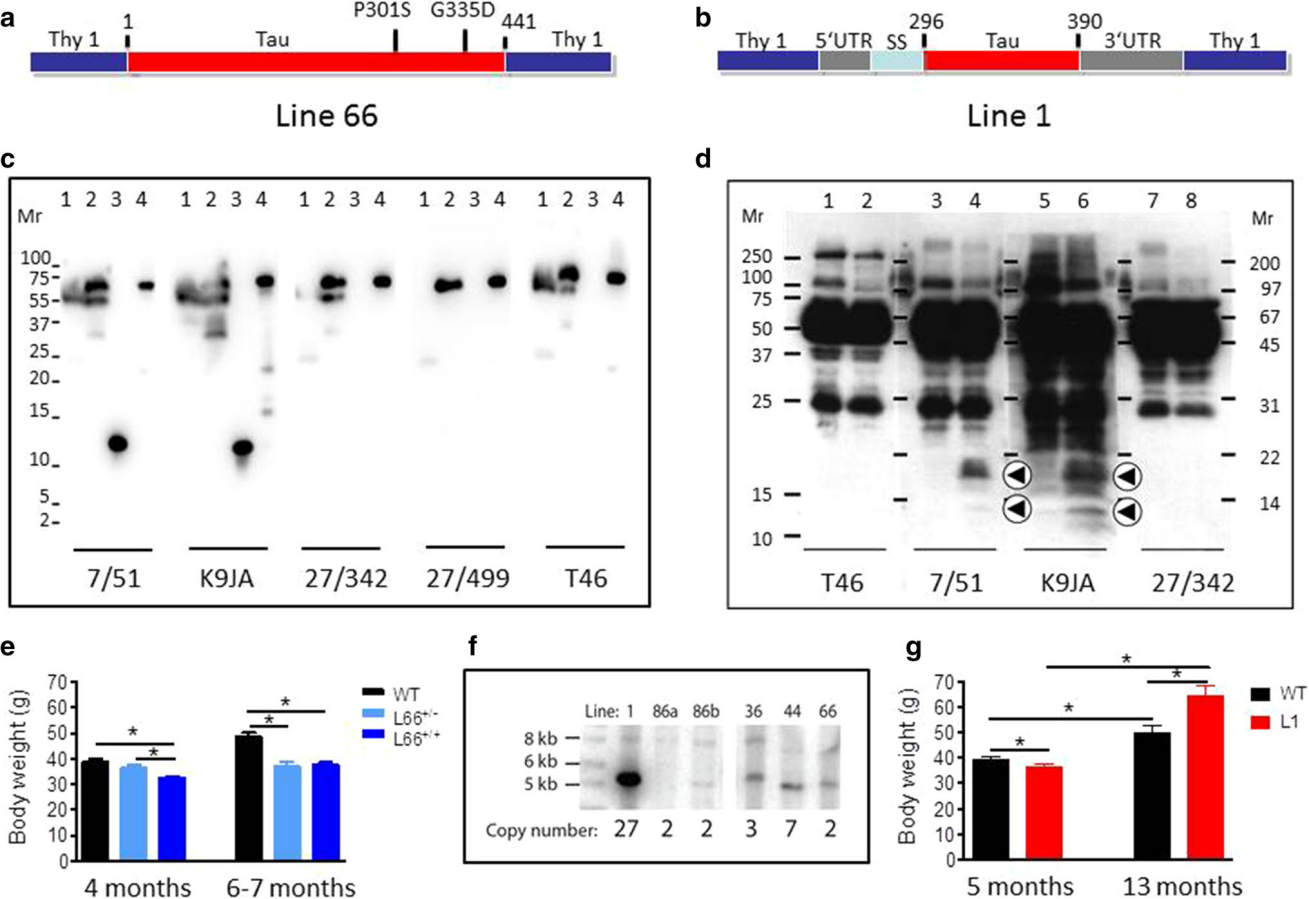
Constructs used for microinjection and expression parameters of protein for full-length mutant and truncated tau. **a** The L66 cDNA construct contains human tau cDNA coding for the longest human CNS tau isoform (htau40; 441 amino acids) with the point mutations to make the amino acid changes P301S and G335D. **b** The L1 cDNA construct contains human tau cDNA coding for amino acid residues 296–390 with signal sequence and related sequences in murine Thy-1 expression cassette. **c** Immunoblot analysis of 6-month-old WT (*lane 1*) and L66 (*lane 2*) mouse brains with recombinant truncated dGAE (*lane 3*) and full-length htau40 (*lane 4*) for reference. The faint 25-kDa band seen in *lane 1* was only seen with the wild-type mouse brain and was observed when the secondary anti-mouse antibody was used in the absence of primary tau antibody. The 70-kDa band reflects full-length mutant tau and is detected by all of the antibodies shown. Note that the 55-kDa band, that is not detected by the human-specific mAb 27/499, represents endogenous mouse tau. The position of markers indicates relative molecular mass (*M*_r_) in kDa. **d** Immunoblot characterisation of tau in 4-month-old L1 (*even numbers*) and WT mice (*odd numbers*). Protein extracts from whole-brain lysates were separated by SDS-PAGE and immunoblots stained with antibodies 7/51 and K9JA for epitopes within the repeat domain of tau. Immunoreactive bands at 12- and 18-kDa (*arrowheads*) were detected in L1 mice (*lanes* *2*, *4*, *6* and *8*) but not in extracts from wild-type animals (*lanes 1*, *3*, *5* and *7*). Antibodies for C-terminal (T46) and N-terminal (27/342) epitopes did not react with these proteins. The intense bands at 25- and 50-kDa reflect endogenous mouse antibody chains detected by secondary antibody. **e** Reduced body weight in female L66 cohorts (wild-type: *n* = 12 at 4 months; *n* = 24 at 6–7 months. L66^+/−^: *n* = 17 at 4 months; *n* = 13 at 6–7 months. L66^+/+^: *n* = 17 at 4 months; *n* = 25 at 6–7 months). Mean + SE; *p < 0.05, *t* test. **f** Southern blot to determine transgene copy number, using genomic DNA of heterozygous founder mice of each line. Hybridisation with DNA of approximately 5 kb corresponds to the transgene, and 8.2 kb to WT tau. *Lines 36*, *44* and *66* carry the mutant full-length tau construct (see in **a**); *Lines 1*, *86a* and *86b* carry the short construct (see in **b**). The numbers of DNA copies incorporated into the genome for each line are given. **g** Reduced weight at young and increased weight at old age in female homozygous L1 mice (WT: *n* = 42 at 5 months; *n* = 9 at 13 months; L1: *n* = 45 at 5 months; *n* = 15 at 13 months). Values expressed as mean + SE; *p < 0.05, *t* test

The construct used for L66 (Fig. 1a) comprises the longest tau isoform in the central nervous system (htau40; 441 amino acid residues) with four repeats and containing the point mutations P301S and G335D. This was cloned into the Thy-1 expression cassette and administered through pronuclear injection. Founder lines (Fig. 1f) showed various copy numbers of transgenes. Two of these were examined further, Lines 36 (L36) and L66. The strongest pathology was seen in L66 mice (e.g., see comparison with Line 36, see Fig. 5d, e). Expression of mutated full-length proteins was confirmed in brain homogenates by immunoblotting which showed a protein with gel mobility corresponding to 70 kDa. This was recognised by a panel of antibodies having epitopes spanning the length of the protein (Fig. 1c), including a human-specific N-terminal epitope recognised by mAb 27/499. By contrast, endogenous mouse tau with gel mobility corresponding to 50 kDa did not react with mAb 27/499.

Offsprings of L66 were healthy and developed normally after birth. We did not observe any behavioural abnormalities upon cage-side assessment. However L66 mice showed lower body weights by 4–7 months of age and this reduction was more pronounced in homozygous mice (Fig. 1e) compared with heterozygous mice. Homozygous female mice frequently developed a palsy-like dyskinesia that was apparent on inspection by the age of 6–7 months. This did not curtail life expectancy or affect other daily activities.

#### Immunohistochemical, neuropathological, ultrastructural and biochemical characterisation of L66 mice

**Fig. 2:**
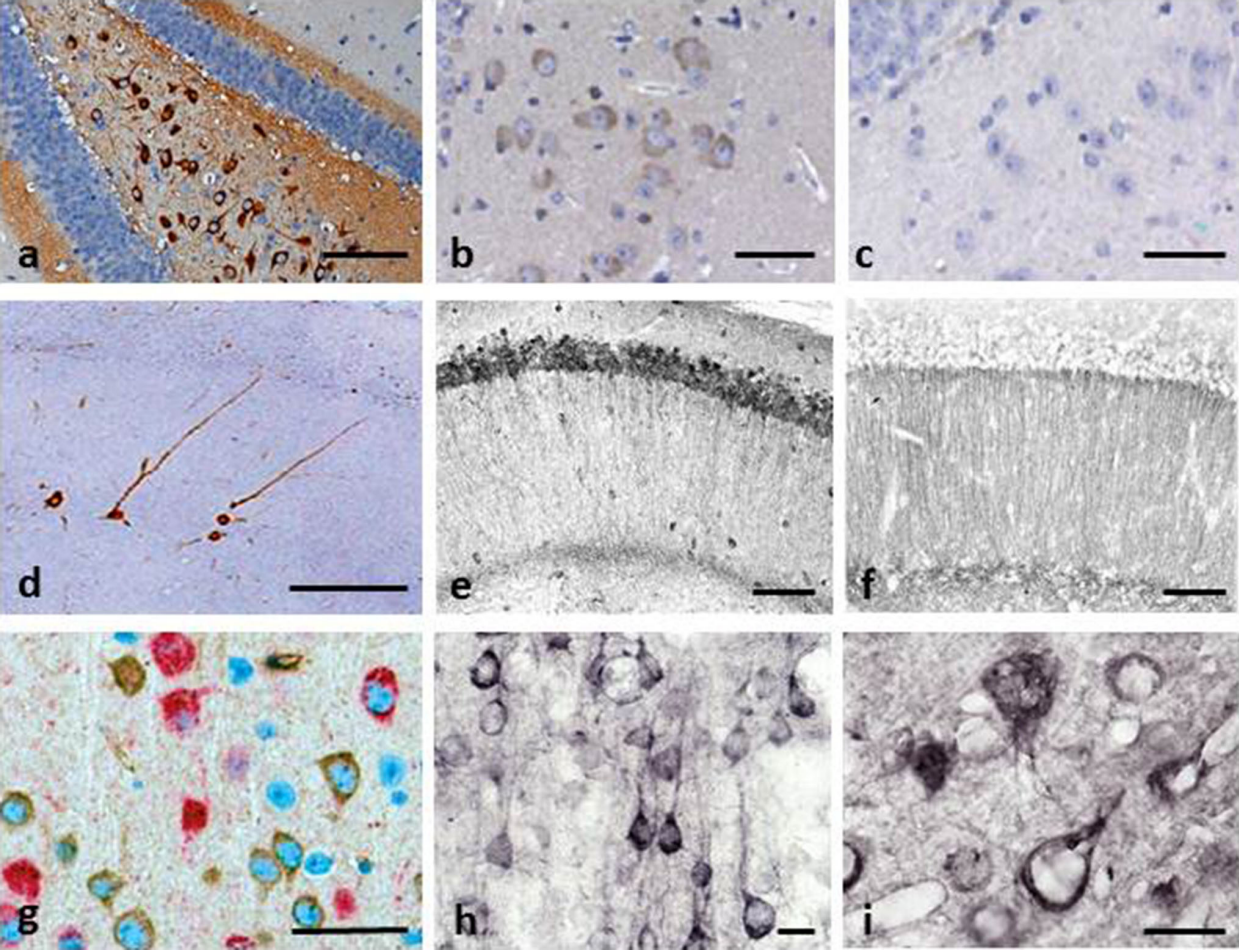
Tau immunohistochemistry of transgenic tau L66 (**a**, **b**, **e**, **f**, **i**) and L1 (**c**, **d**, **g**, **h**) mice. Tau immunoreactivity with mAb 7/51 is strong in L66 with prominent intracellular staining in the hilus/CA4 sectors of the hippocampus (**a**) and in other areas (see Fig. 4). In contrast, mAb AT8 immunoreactivity is seen more frequently in elderly mice with staining apparent in long processes of CA1 (**d**). Neurons in visual cortex (**g**) are stained differentially with both mAb 7/51 (*brown*) and AT8 (*red*). Amorphous intracellular tau immunoreactivity, visualised using mAb 7/51, in the hilus/CA4 of L1 mice (**b**) is much weaker than for L66, and absent from the corresponding region in WT mice (**c**). The presence of pS404 phospho-tau immunoreactivity was seen in hippocampus and cortex and was more intense for L1 late in life. At 3 and 9 months old, L1 and WT animals were similar. It was only at 15 months of age that a prominent difference between L1 and wild-type was observed, with CA1 labelling for pS404-Tau greater in L1 (**e**) than WT (**f**). The staining of neurons with this mAb indicates that there are granular accumulations of pS404-Tau immunoreactivity in long processes (**h**, **i**). *Scale bars* 50 µm (**a**, **d**); 25 µm (**e**–**g**); 20 µm (**b**, **c**) and 10 µm (**h**, **i**)

Brain tissue from both L36 and L66 mice had clearly identifiable intracellular tangle-like tau immunoreactivity using mAb 7/51 (which recognises an epitope in the repeat domain) and mAb AT8 (which recognises a phosphorylation-dependent conformational epitope). These features were seen in both cortical (Fig. 2a: CA1 and hilus) and non-cortical (see Fig. 4 below) regions with both antibodies. In addition, punctate staining in neuronal cell bodies, extending into the proximal part of their axons (Fig. 2g), was observed in L66 but not in L36. No staining was seen with either antibody in any region in wild-type mice. That is, although mAb 7/51 recognises an epitope in the repeat domain which is also present in native mouse tau, the antibody labels only pathological aggregates in both AD brain tissues [38, 39] and in transgenic mouse models but not in wild-type animals. Staining of normal tau in wild-type brain tissue requires special fixation conditions [47].

Minimal to mild staining of the CA1, CA3 and hilar neurones was observed with 7/51, but only few hilar neurones were stained with AT8 (not shown). By contrast, axonal staining was much more apparent with mAb AT8 (Fig. 2d). This was present multifocally within the brains of all L66 mice. Many axons were characterised by a discontinuous pattern of staining, suggesting a process of axonal fragmentation.

Although the regions stained with mAbs 7/51 and AT8 were similar, there was also evidence of dissociation with respect to neurons labelled and the region of the neuron labelled. This is shown for example in Fig. 2g where double-labelling has been used to demonstrate the existence of distinct neuronal populations labelled, respectively, with mAb AT8 and mAb 7/51, with little overlap. Therefore phosphorylation of tau, as seen with mAb AT8, is not a necessary requirement for tau aggregation detected using mAb 7/51 either in terms of neurons labelled, or the location within the neuron where labelling occurs.

**Fig. 3:**
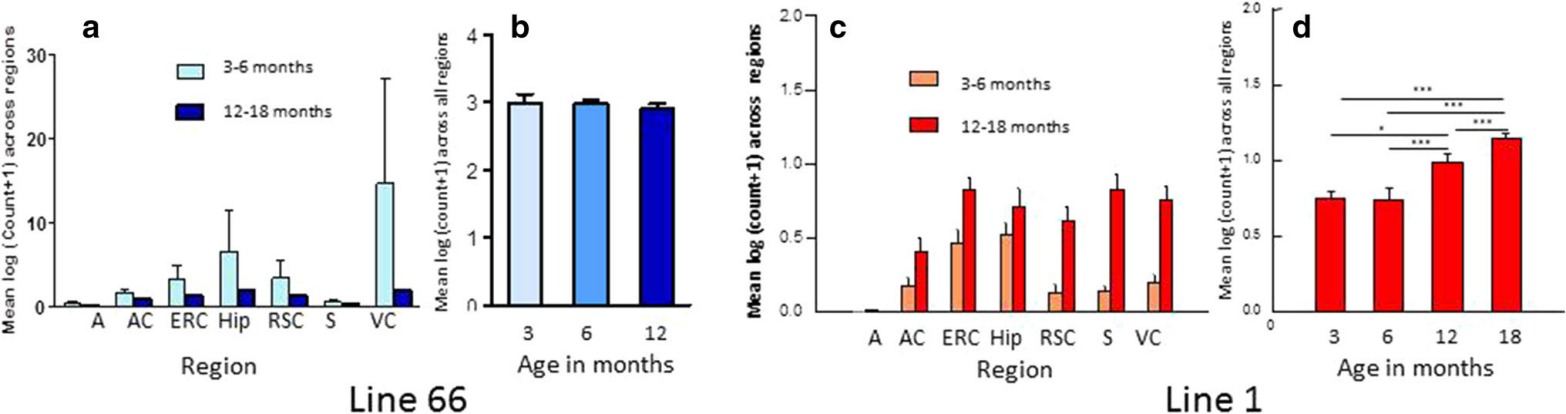
Quantitative regional and age-related tau immunohistochemistry of transgenic L66 and L1 mice. Tau-positive neurons reacting with mAb 7/51 were quantified in homozygous L66 mice by region (**a**) and by age (**b**). Data are presented as group mean log_10_ of the sum across individual brain regions taken from Bregma −3.16 mm according to the stereotaxic mouse atlas [43]. Young animals aged <6 months presented with stronger immunoreactivity in all regions. The cell count (group mean log_10_ + SE) across all regions is already high at 3 months (*n* = 2) and decreases substantially at 6 (*n* = 4) or 12 months (*n* = 5). *A* amygdala, *AC* auditory cortex, *ERC* entorhinal cortex, *Hip* hippocampus, *RSC* retrosplenial cortex, *S* subiculum, *VC* visual cortex. In contrast, for L1 mice (*n* = 6 for each age group) there was a significant age-related increase in labelled cells in all regions in older mice (**c**). Global cell count (group mean log_10_) averaged across all regions (+SE) selected from the same level and plotted as a function of age (*n* = 3 each). There was no difference in tau immunoreactivity in animals aged 3 or 6 months, but pathology increased at 12 and more so at 18 months (*p < 0.05, ***p < 0.001) (**d**). There were approximately sevenfold more tau-positive neurons in L66 relative to L1

Surprisingly, regional counts of histologically positive neurones (using mAb 7/51) were greater in L66 at ages less than 6 months than in mice aged 12 months or more (Fig. 3a). The reasons for this observation are not clear but confirm the absence of age-dependent spread of pathology similar to Braak staging.

**Fig. 4:**
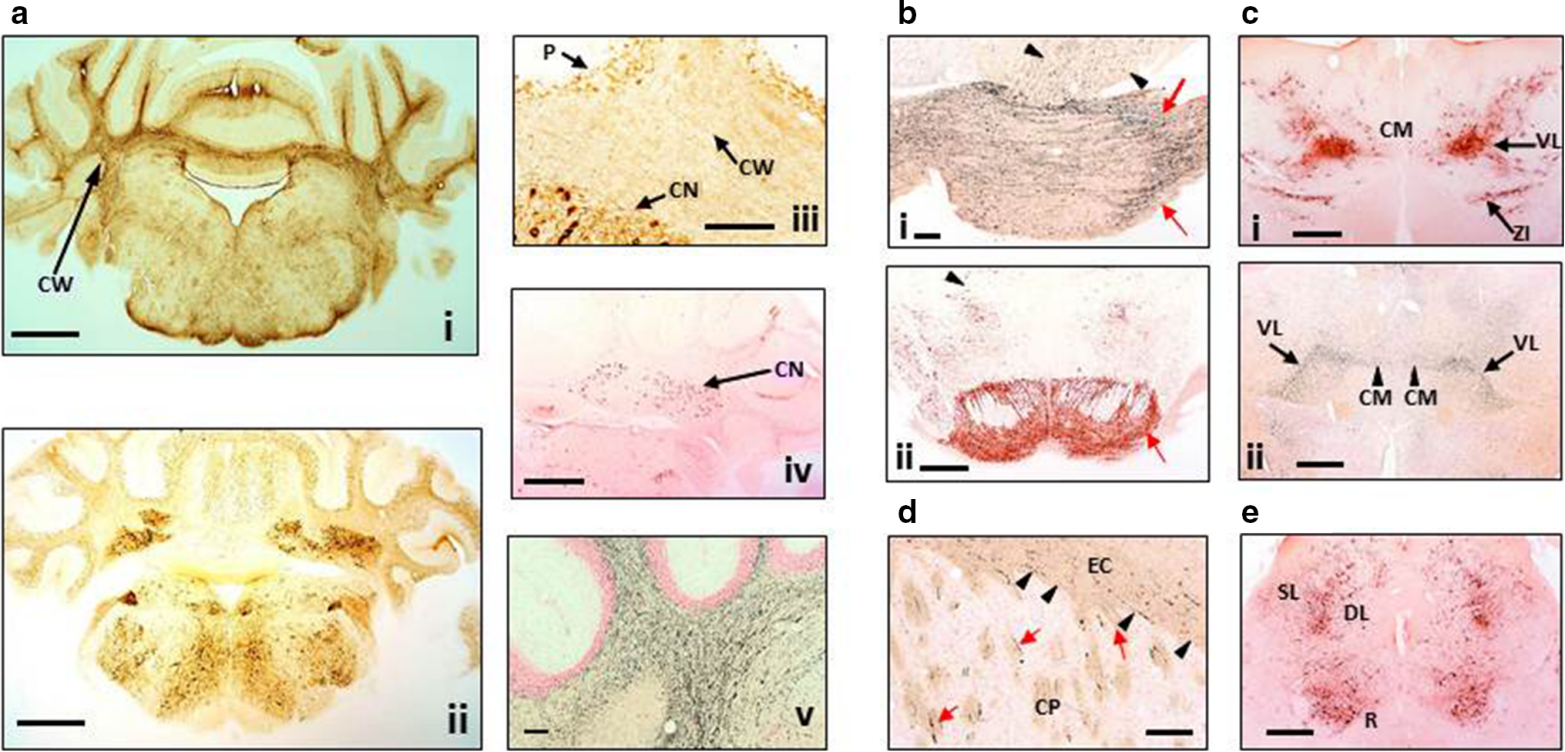
Distribution of aggregated tau in L66 transgenic mice. **a** Staining for astrocytes and axons in the cerebellum of L66 mice aged 6–7 months. **a**-**i** Cross-sections through the mid-cerebellar region and underlying brainstem stained for GFAP in L66 shows immunoreactivity within the cerebellar white matter (CW). Cellular staining for phospho-tau using mAb AT8 (**a-ii**) was identified in both the cerebellar cortex and in the deeper cerebellar nuclei (**a**-**iii**, CN), but also some weak staining in processes running through the white matter, and non-specific immunoreactivity in the Purkinje cell layer (P). Deep nuclei were stained also with mAb 7/51, but no processes were immunopositive (**a**-**iv**). Axons stained darkly by the amino cupric silver stain indicate injury to axonal integrity in L66 mice (**a**-**v**). **b** Degeneration of fibres and expression of human tau in pons and ventral tegmentum. Axonal injury was observed in pons using amino cupric silver staining (**b**-**i**). While *black arrows* indicate scattered positivity for longitudinally oriented axons in tegmental regions, strong labelling of transverse fibres (*red arrows*) occurred in the cerebral peduncle. Somata/processes were also densely labelled for phospho-tau using AT8 (**b**-**ii**) in both pontine axons and cells (*red arrow*) and tegmental neurons (*arrowhead*). **c** Anterior thalamic expression of tau. Dense AT8 labelling (**c**-**i**) was observed in cells and axons of the ventrolateral thalamic nucleus (VL) and zona incerta (ZI) with more scattered immunoreactivity in the centro-medial nucleus (CM) concentrated to the cell soma. Phospho-tau labelling overlapped with axonal injury as revealed by amino cupric silver staining (**c**-**ii**) and was most clearly visible in VL and CM. **d** Basal ganglia axonopathy in L66. The coronal section is stained with amino cupric silver and magnified to reveal axonal degeneration in both the external capsule (EC; *arrowheads*) and caudate-putamen (CP; *arrows*). **e** Low magnification micrograph of the cross-section through the L66 midbrain labelled with AT8. Prominent cellular and axonal phospho-tau labelling occurred in the red nucleus (R) and deeper layers (DL) of the superior colliculus with processes reaching towards the dorsolateral superficial layers (SL). *Scale bars* **a-i**, **a-ii** 1 mm; **a-iv**, **b**-**ii**, **c-i**, **c-ii**, **e** 0.5 mm; **a-iii**, **a-v**, **b-i**, **d** 100 mm

Labelling with mAb AT8 in L66 was also associated with axonopathy. Staining in frontal cortex was more prominent than piriform cortex, parietal regions and amygdala (not shown). Cerebellar axonopathy was evident in cerebellar cortex and deep nuclei sections of L66 (Fig. 4a-i, iii), but also showed neuronal staining with mAb AT8 (Fig. 4a-ii). No axonopathy in deep cerebellar nuclei was found with mAb 7/51 (Fig. 4a-iv). Phospho-tau mAb AT8 labelled neurones in both pontine axons and cells (Fig. 4b-ii), but also more anterior thalamic nuclei including the ventromedial thalamic nuclei and zona incerta (Fig. 4c-i) including subthalamic nuclei, the red nuclei and various layers of the superior colliculus (Fig. 4e).

Similarly widespread was the amino cupric silver-positive staining for axonal disintegration encompassing the frontal cortex and the external capsule, the white matter of the caudate-putamen and the internal capsule (Fig. 4d). White matter degeneration was particularly strong in the lateral reticular nucleus of the rostral thalamus (not shown), but also in the ventromedial thalamic nuclei extending into the central portion of the thalamus (Fig. 4c-ii). Staining was weaker in caudal thalamus and rostral midbrain, mainly present in the medial lemniscus and adjacent cerebellar peduncles. Within the caudal midbrain, strong axonal staining was present in transverse fibres of the pontine reticular region (Fig. 4b-i). At the level of the cerebellum, prominent axonal cupric silver staining was present in the deep cerebellar white matter (Fig. 4a-v), the acoustic nerves and cochlear nuclei, the spinal trigeminal tract and within the medulla oblongata.

**Fig. 5:**
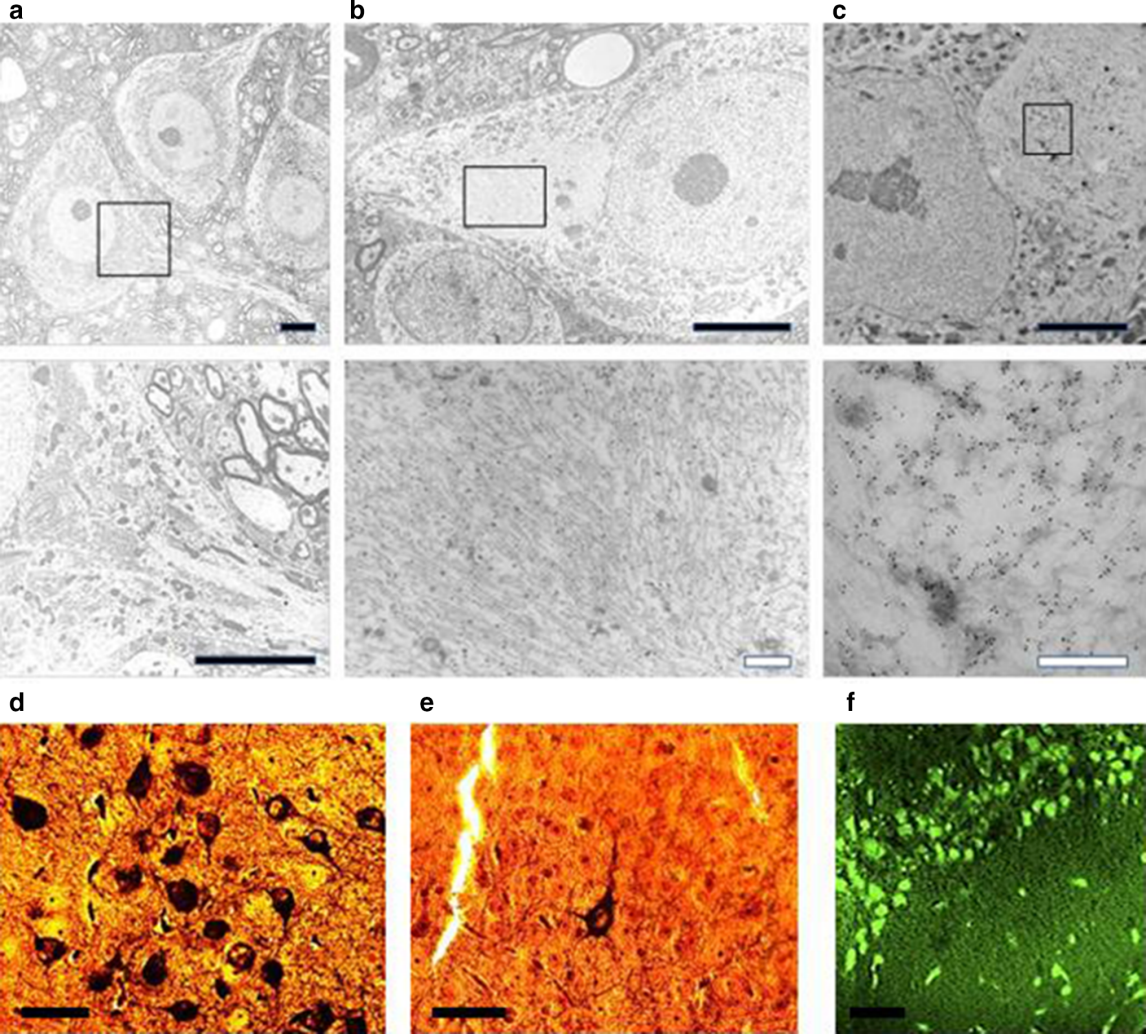
Filamentous tau in L66. Electronmicroscopy of spinal cord from **a** WT and **b** L66 mice and **c** anti-tau immunoelectronmicroscopy of L66. Dense bundles of filaments were found in large neurons in L66 but not WT mice and these were immunodecorated with gold particles labelling tau. The *lower panel* of images are selected magnifications from the *upper panel* as indicated; *scale* 5 µm (*solid bars*) and 0.5 µm (*open bars*). Bielschowsky silver staining for tau aggregates was more abundant in cortical sections from L66 (**d**) than L36 (**e**) mice. In addition, Primulin-stained neurons provide further evidence of the formation of filamentous tau aggregates in cortical tissue from L66 (**f**)

Electronmicroscopic (EM) investigations were undertaken in spinal cord from L66 mice because other tissues were used for quantitative immunohistochemistry and neuropathological investigations. EM revealed the accumulation of abundant pathological filaments in L66 mice aged 8–9 months (Fig. 5b, c), that were absent from age-matched wild-type mice (Fig. 5a). The appearance and distribution of cytoplasmic organelles in the cell bodies appeared normal and the pattern of axonal microtubules was regular in wild-type mice (Fig. 5a). In contrast, large filamentous aggregates were found in neuronal cell bodies in L66 that displaced organelles such as mitochondria, Golgi apparatus and endoplasmic reticulum (Fig. 5b). Areas containing filamentous material were strongly labelled with immunogold particles using a polyclonal anti-tau serum that does not show phosphorylation-dependent immunoreactivity. By contrast, there were only occasional gold particles detected in cytoplasmic and nuclear areas without filaments (Fig. 5c), and there was no immunogold labelling in wild-type mice (not shown). The pathological filaments formed in L66 brain tissues are biochemically PHF-like in that they contain a proteolytically stable core restricted to the repeat domain comprising only 3 repeats [1, 4, 48].

Both Bielschowsky silver and primulin staining confirmed that tau derived from mutant full-length transgenes aggregates into filamentous structures in L66 (Fig. 5d–f) and with a higher density than seen in L36 (Fig. 5e). Intracellular aggregation was found in the somatodendritic compartment of neurons.

#### Prominent age-dependent impairment in sensorimotor performance and motor learning in L66

**Fig. 6:**
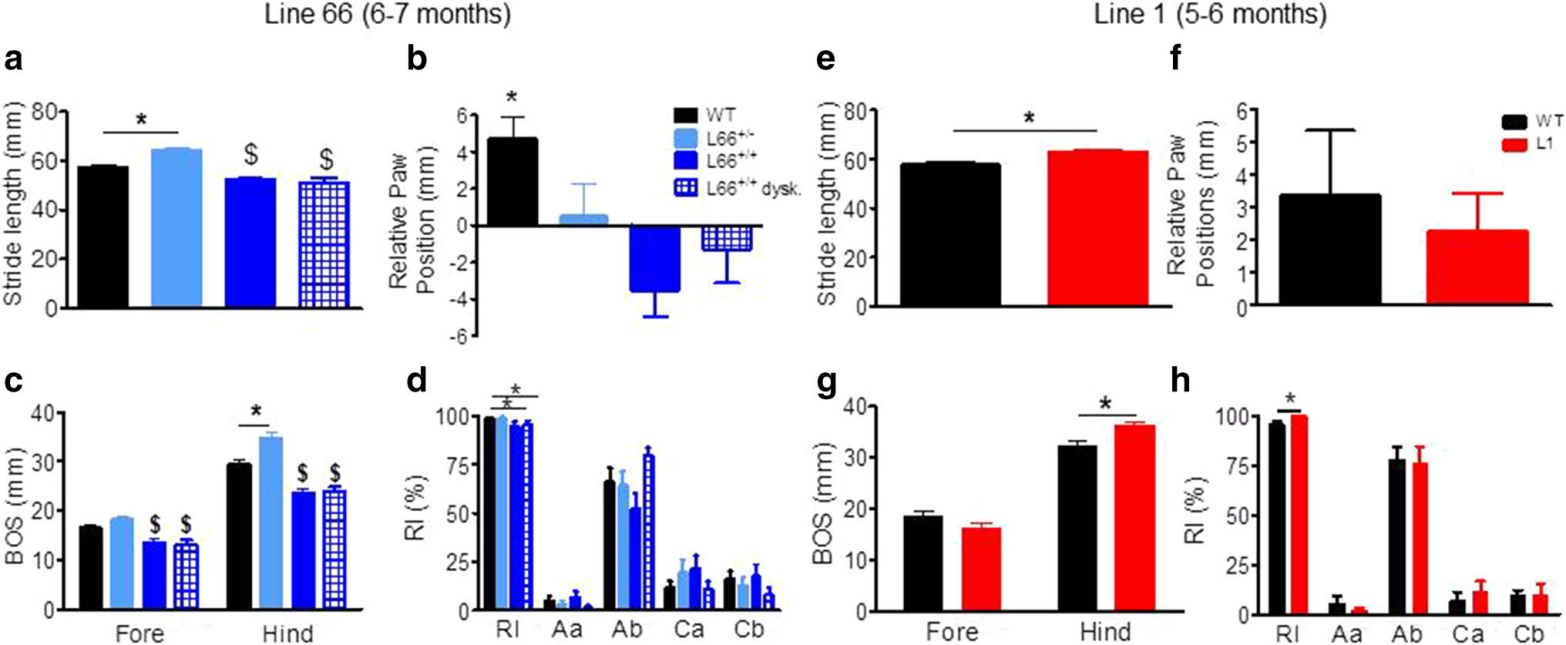
Gait analysis of transgenic L66, L1 and wild-type mice using the CatWalk. *Left* Aged 6–7 months, four different female groups of L66 were tested: (1) WT, *n* = 24; (2) L66^+/−^, *n* = 14; (3) L66^+/+^ with dyskinesia, *n* = 12; (4) L66^+/+^ without dyskinesia, *n* = 13. Note that animals with palsy-like dyskinesia did not perform different from non-dyskinetic littermates. While heterozygous L66 mice presented with longer stride length, L66^+/+^ mice had reduced length of footfall with no difference between front or hind paws (**a**). WT showed the NMRI-typical positive overlap with hind paws falling behind forepaws, L66 had generally no overlap (**b**) or fell significantly in front of forepaw (a negative value implicates that the hindpaw is placed upward with respect of the forepaw and conversely, a positive value indicates that the hindpaw is placed backward compared to the forepaw). In general, all genotypes had greater base of support (BOS) for hindlimbs and, while heterozygote mice tended to have wider gaits, homozygous groups brought front and hind paws closer together (**c**). This may have led to a lowering in the regularity index (RI). The RI grades the degree of coordination and represents the percentage of regular step patterns in which the paws are placed on the glass plate—alternate patterns (i.e. *Aa* and *Ab*) and cruciate patterns (i.e. *Ca* and *Cb*) (**d**). *Right* Homozygous L1 female mice and WT were compared (*n* = 14 each) at 5–6 months. Stride length (mean of front and hind paws) was greater in L1 mice (**e**), but both WT and L1 placed hind paws behind the front paws (**f**: positive values). The overall base of support was not affected for front, but higher for L1 hind paws (**g**). A small increase in RI (**h**) but no significant difference in axial or cruciate paw placements was revealed. All data expressed as group mean + SE; *p < 0.05, *t* test. ^$^Difference from WT and L66^+/−^ groups

We observed numerous abnormalities with respect to gait and walking patterns in L66 mice (Fig. 6). L66 animals differed from wild-type mice in stride length (Fig. 6a; *F*(3, 123) = 16.8, *p* < 0.0001) with heterozygous and homozygous mice having longer and shorter strides, respectively. A similar difference occurred for the base of support for both front and hindlimbs (Fig. 6c: *F*(3, 50) = 11.6, *p* < 0.0001 fore; *F*(3, 50) = 26.3, *p* < 0.0001 back). Relative paw position showed normal positive values in wild-type mice but not L66 mice (Fig. 6b; *F*(3, 123) = 6.2, *p* < 0.0006). A marginal reduction in regularity index was seen in homozygous L66 mice (Fig. 6d; *F*(3, 61) = 2.6, *p* = 0.06) but no difference occurred for parameters related to step cycle (data not shown). Surprisingly, L66 mice with prominent dyskinesia did not show greater evidence of gait disturbance on these parameters compared to those without.

**Fig. 7:**
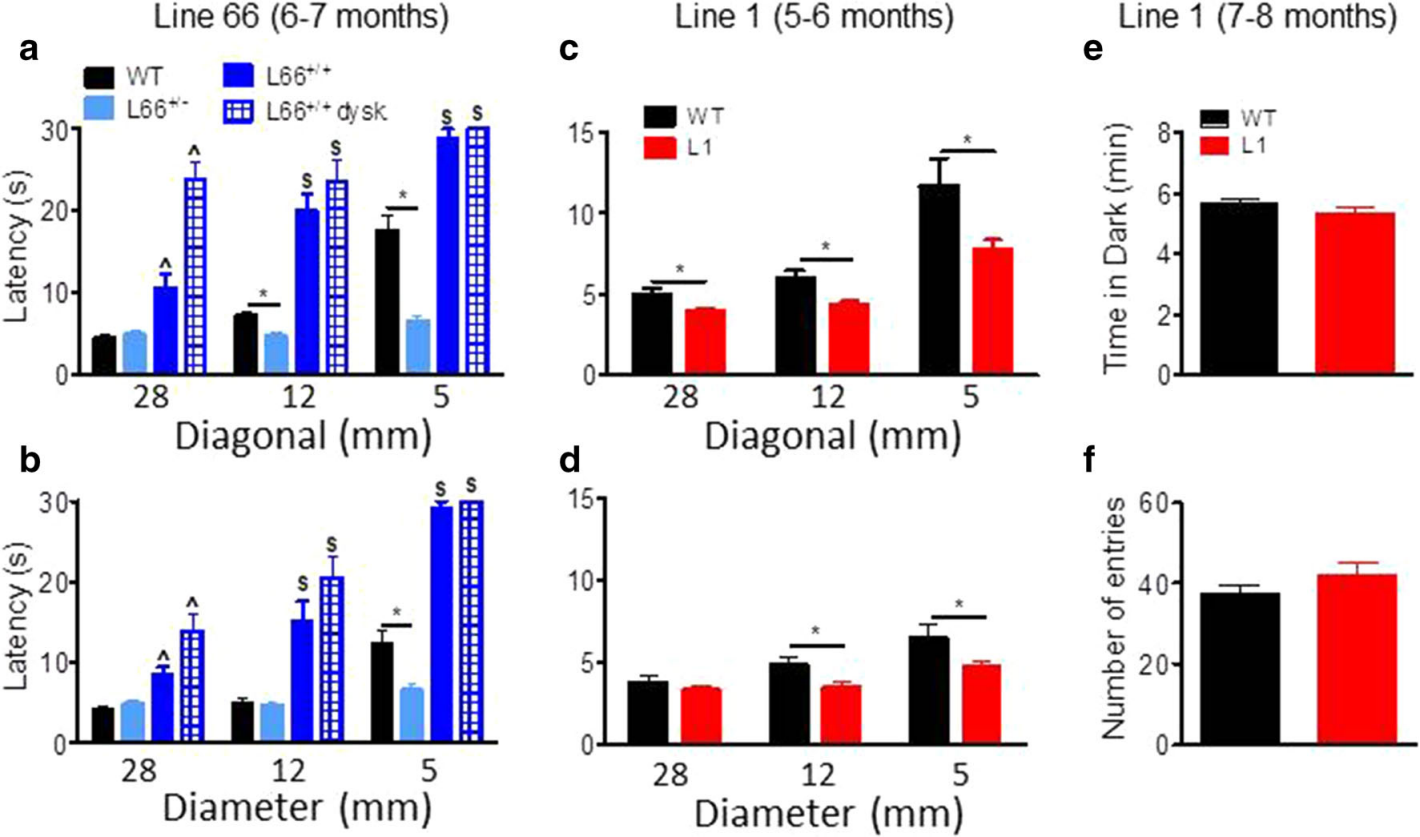
Differential phenotypes for L66 (**a**, **b**) and L1 (**c**, **d**) on the balance beam and in the light/dark box tests (**e**, **f**). For heterozygous L66 mice (6–7 months), while performing equally or better than age-matched controls at 5- or 12-mm beams, deficiencies were observed in homozygous females, which were impeded at all beams (**a**, **b**). Note that animals with dyskinesia performed worse than normal age-matched homozygous mice (WT *n* = 24; L66^+/−^ *n* = 14, L66^+/+^ *n* = 25). For 5- to 6-month-old L1 mice, latency to traverse square beams was shorter (better performance) than wild type (*n* = 14 each, females) (**c**). There was a similar finding for L1 with round beams (**d**). Increase in latency for the more difficult beams reflects the more challenging nature of small beams. In the light/dark box test, L1 and control mice aged 7–8 months (WT *n* = 13; L1 *n* = 13) spent similar amounts of time in the dark compartment (**e**) and presented with comparable number of entries (**f**). Values expressed as mean + SE; *asterisks* denote group differences (Student’s *t* test) at *p* < 0.05. ^^^Statistically significant difference from all the other groups; ^$^difference from WT and L66^+/−^ groups

On the balance beam, heterozygous L66 mice performed similarly or better than wild-type controls (shorter latencies for reaching the end of the beams). By contrast, homozygous L66 mice were severely impaired (Fig. 7). Furthermore, performance on the balance beam was significantly worse in homozygous animals with dyskinesia than in those without. These effects were observed for both square (Fig. 7a; effect of genotype, beam size and interaction: *F* > 9, *p* < 0.0001) and round beams (Fig. 7b; effect of genotype, beam size and interaction: *F* > 11, *p* < 0.0001). Pairwise comparisons (using the Bonferroni correction for multiple comparisons post hoc) yielded numerous statistically significant differences with respect to controls (see asterisks in Fig. 7a, b).

**Fig. 8:**
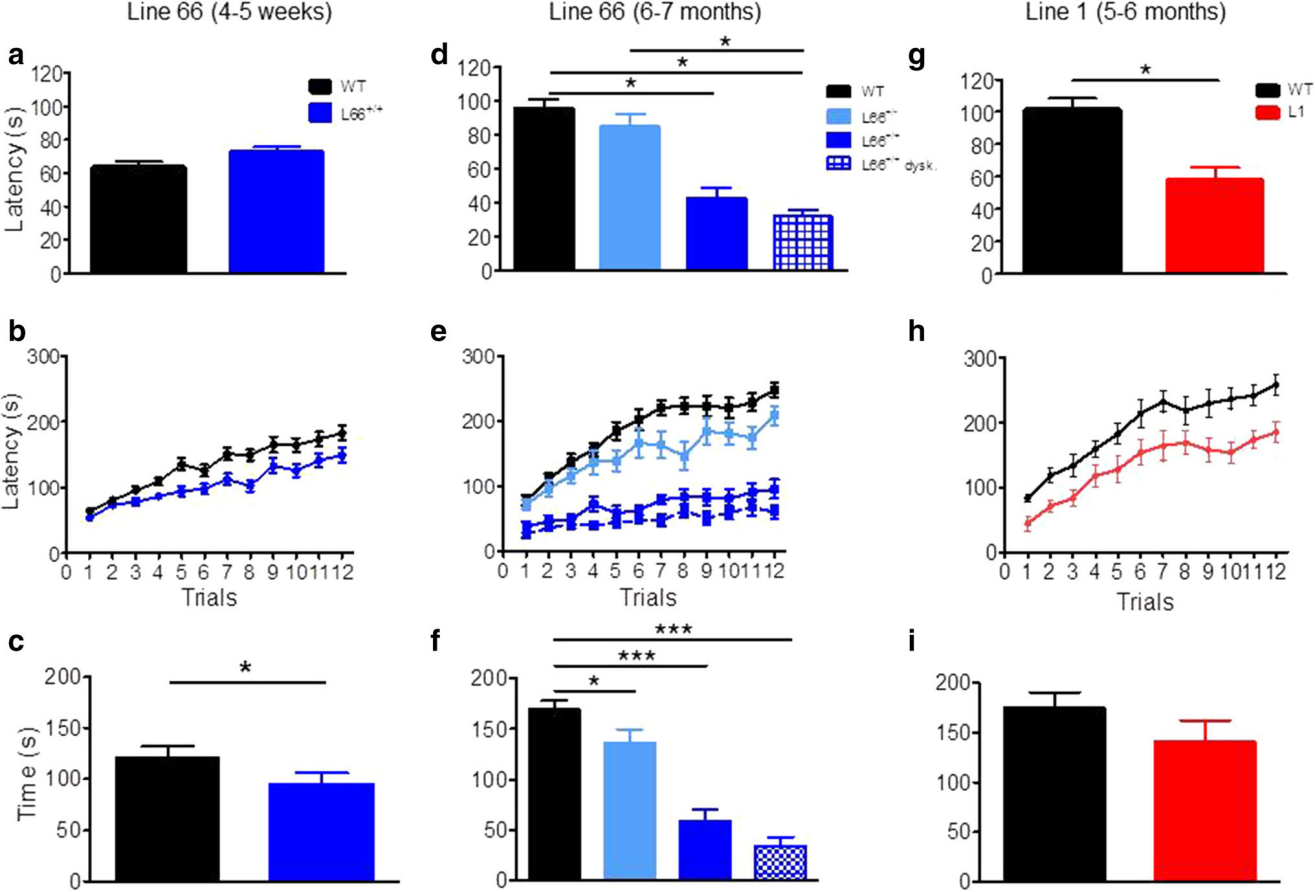
Female tau mice show deficits in motor coordination and motor learning. *Top row* (**a**, **d**, **g**) Latency to stay on the accelerating RotaRod in Trial 1 and 2 as an index for motor coordination. L1 mice (**g** 5–6 months; WT *n* = 14; L1 *n* = 14) were significantly impaired, as were homozygous L66 (**d** 6 months; WT *n* = 24, L66^+/−^ *n* = 14; L66^+/+^ *n* = 13; L66^+/+^ dysk. *n* = 12), but not L66 aged 4–5 weeks (**a** WT *n* = 40; L66 *n* = 34). Heterozygous L66 did not present with a phenotype up to 6 months. *Middle row* (**b**, **e**, **h**) Motor learning on the RotaRod over 12 trials (3 days of 4 trials) was impaired in all transgenic cohorts including L1 (**h**), as well as homo- and heterozygous L66 at 4–5 weeks (**b**) or 6 months (**e**). *Bottom row* (**c**, **f**, **i**) Overall performance gain between trial 1 and 12 in different cohorts. Despite a performance deficit in L1, the overall amount of improvement was not different to controls (**i**). Similarly, all L66 cohorts had significant learning different from 0 (not indicated), but L66 mice typically improved less than WT both at 4-weeks (**c**) and 6 months (**f**). Values expressed as mean ± SE; *asterisks* denote group differences (Student’s *t* test) at *p* < 0.05

Motor performance (Trials 1 + 2) was also investigated on the RotaRod (Fig. 8). Whereas homozygous mice aged 4–5 weeks were not impaired in motor performance (Fig. 8a), by 6 months of age they showed impairment (Fig. 8d; effect of genotype: *F*(3, 125) = 22.5, *p* < 0.0001; see asterisks for pairwise comparison with wild-type). The level of impairment was not greater in homozygous L66 mice with dyskinesia compared to those without. Motor learning was already impaired in homozygous L66 mice aged 4–5 weeks (Fig. 8b; genotype x trial interaction: *F*(11, 792) = 1.9, *p* = 0.04), and this deficit increased considerably in 6-month-old cohorts (Fig. 8e; interaction: *F*(33, 649) = 4.4, *p* < 0.0001). Six-month-old heterozygous mice were mildly impaired (*F*(11, 396) = 1.6, *p* = 0.01). In homozygous L66 mice (Fig. 8d–f), the presence of dyskinesia was associated with further enhancement of the deficit (dyskinesia effect: *F*(11, 253) = 0.9, *p* = 0.05).

In addition, we assessed the amount of the learning deficit at the end of training by comparing the difference in improvement between first and last training trials. We reasoned that severe learning deficits (for instance in homozygous L66 mice aged 6 months) may preclude any improvement relative to start of training. While all cohorts showed significant improvements in performance in trial 12 relative to trial 1 (Fig. 8c, f; all *p* values <0.01 when compared to 0), we established considerable differences in the amount of learning between cohorts (see asterisks). While heterozygote L66 mice presented with small impairments at 6 months, deficits were more severe in homozygous groups. Again, dyskinesia did not enhance severity of the deficit relative to non-dyskinesic age-matched subjects (Fig. 8f).

#### Absence of cognitive-spatial deficits in L66 mice

**Fig. 9:**
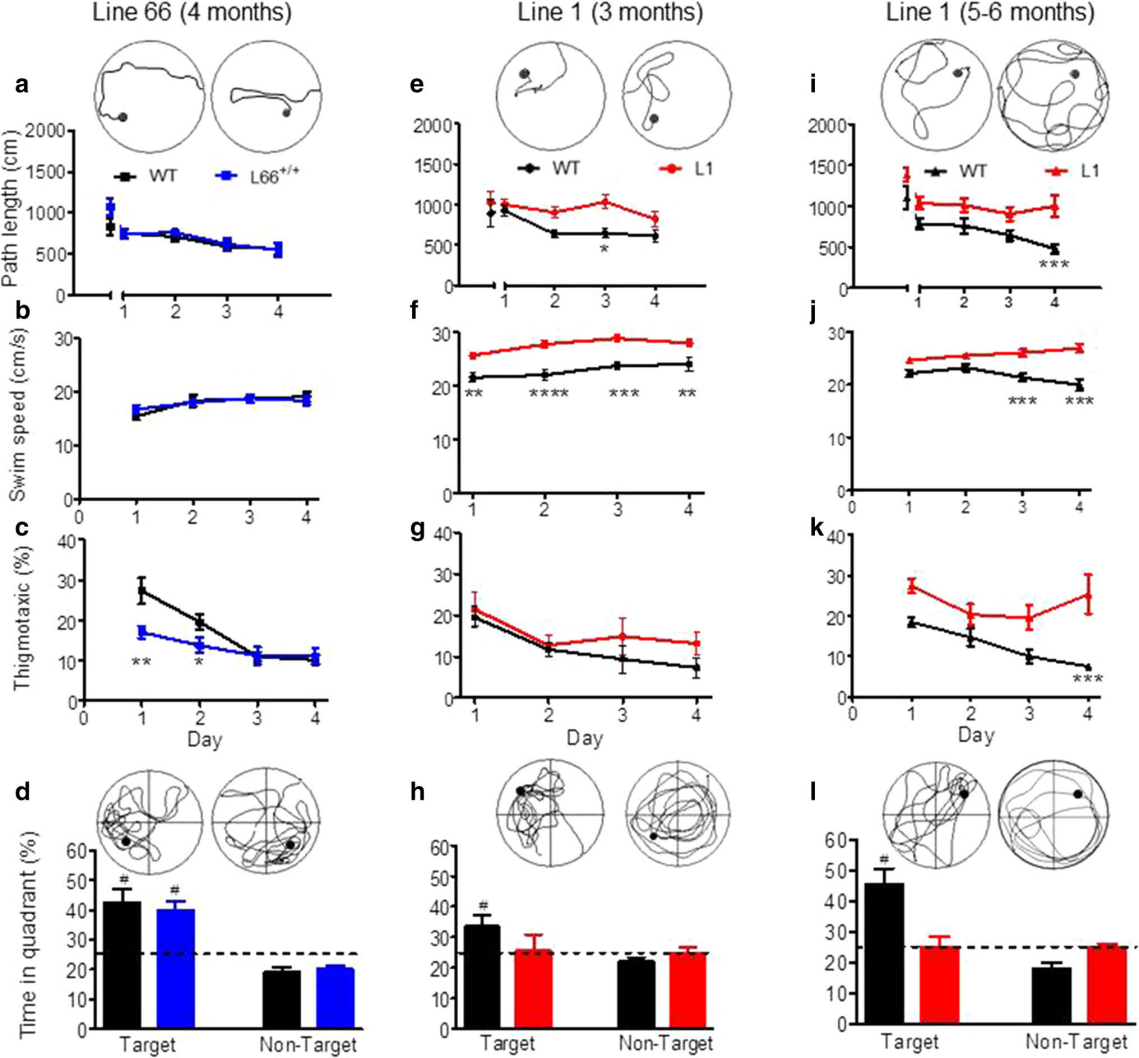
Spatial learning deficits are absent in L66 transgenic mice but observed in L1. Standard open field water maze acquisition learning and recall are shown for L66. No spatial phenotype was observed in L66^+/+^ mice aged 4 months (**a**–**d** WT *n* = 12; L66^+/+^ *n* = 13). There were no differences in path length (**a**) and swim speed (**b**), but lower levels of thigmotaxis occurred on the first 2 days of training (**c** see *asterisks* for *p* < 0.05). Also, there was no deficit in recall indexed as quadrant time (**d**). Standard open field water maze acquisition learning and recall are shown for cohorts of L1 mice aged 3 months (**e**–**h** WT *n* = 9; L1 *n* = 10) and 6 months (**i**–**l** WT *n* = 10; L1 *n* = 13). Three-month-old L1 mice need longer swim paths to find the hidden platform (**e**), but eventually acquire the spatial task after 24 trials (day 4). Note that there was no difference in path length in trial 1, but a strong retardation in learning. Young L1 mice swam faster throughout acquisition (**f**), but thigmotaxis was not significantly enhanced (**g**). Recall (time in quadrant) tested 1 h post-training was deficient in L1, but there was a weak tendency for the target quadrant in controls (**h**). Deficits in 6-month-old L1 mice were more pronounced. Transgenic mice failed to acquire the spatial task (**i**), swam considerably faster (**j**) and maintained high levels of thigmotaxis (**k**) relative to age-matched controls. Consistent with the failure to acquire the task, there was no spatial bias for the target quadrant in L1 subjects (**l**) whereas controls developed strong memory for the platform location. All data represent mean ± SE. *Asterisks* indicate *p < 0.05, **p < 0.01, ***p < 0.001, ****p < 0.0001 for group comparison; ^#^ *p* < 0.05 relative to chance. *Insets* depict representative swim paths for the last acquisition trial on day 4 (*top row*) and during probe test (*bottom*) for WT (*left*) and L66 or L1 (*right*)

Despite the abundance and severity of pathology seen in L66 mice, no cognitive phenotype was observed in the standard version of the water maze (Fig. 9). Both L66 and wild-type mice swam the same path length to find the platform and attained identical performance on day 4; statistical analysis confirmed learning (main effect of trial day: *F*(4, 88) = 4, *p* = 0.005), but no effect of genotype (Fig. 9a). Moreover, there was no difference in swim speed (Fig. 9b). Although genotype and day interacted with respect to time spent in the thigmotaxis zone (Fig. 9c; *F*(3, 66) = 4.7, *p* = 0.005), this difference was transient and only occurred during the initial 2-day acquisition period. Furthermore, L66 mice presented with a strong spatial bias in the probe trial (Fig. 9d), spending more time in the target quadrant, as was seen also with wild-type controls. Therefore, L66 showed evidence of normal acquisition learning of the standard spatial discrimination task.

### Over-expression of the truncated PHF-core repeat domain fragment of tau in L1 is characterised by non-fibrillar tau pathology associated with a prominent cognitive phenotype and minimal motor features

The truncated repeat domain fragment of tau corresponding to residues 296–391 or its homologues in other tau isoforms is the predominant constituent of the core of the PHFs found in the neurofibrillary tangles of AD. However, constitutive or transient overexpression of this fragment in cells is highly toxic. As reported elsewhere, we have shown that the addition of an N-terminal signal sequence which targets the protein to the ER membrane has the benefit of ameliorating toxicity whilst nevertheless retaining the ability to aggregate, and to both recruit normal tau into the aggregates and induce its template-directed truncation [48]. We used this construct, therefore, to generate the transgenic L1 mouse.

#### General observations

Details of the generation of L1 mice are shown in Fig. 1. The short construct (Fig. 1b) coding for amino acids 296–390 was cloned into a Thy-1 expression cassette and injected into NMRI oocytes. Numerous founders were identified (Fig. 1f) and Southern blot revealed a high number of integrated transgenic copies in L1 (27 copies) relative to all other transgenic lines of this construct. A further line (Line 86b) had integrated 2 transgenic copies. Expression of the truncated tau fragment was confirmed by immunoblotting for L1 only. The transgenic protein was found at low levels in brain homogenate and had gel mobilities corresponding to 12- and 18-kDa. This fragment reacted with repeat domain-specific antibodies mAb 7/51 and K9JA, but not with antibodies recognising epitopes located in the N- or C-terminal domains of tau (Fig. 1d). These 12- and 18-kDa bands were absent in brain homogenates from wild-type (WT) mice.

Homozygous L1 mice had lower body weights at 5 months of age (*t* = 2, *p* = 0.04; Fig. 1g), but gained weight more rapidly to exceed the weight of wild-type mice by the age of 13 months (*t* = 2.6, *p* = 0.01). The animals tended to be hyperactive and showed enhanced freezing in novel environments.

#### Immunohistochemistry of L1 shows age-dependent spread of tau-aggregation pathology similar to Braak staging

Immunohistochemical staining with mAb 7/51 was widespread and mainly localised to neuronal somata in hippocampus (Fig. 2b) throughout cortex, and also in spinal cord and cerebellum (not shown). Line 86b, by contrast, was devoid of labelling even at 18 months and no labelling was seen in wild-type controls (Fig. 2c). The tau immunoreactivity was typically amorphous in character, with no evidence of formation of tangle-like structures. In particular, there was no staining with Bielschowsky silver, primulin or thioflavin S (not shown). Although there was already weak labelling with p-Tau (S-404) (which labels a C-terminal phospho-serine epitope) in wild-type animals, this was greatly accentuated in L1 mice only by 15 months (Fig. 2e, f). The labelling was seen in cortical neurons at 15 months (Fig. 2h, i). No labelling was seen with the phosphorylation-dependent mAb AT8. Therefore, tau aggregation seen in L1 mice was halted at the amorphous/oligomer stage and did not progress to formation of pathological filaments or neurofibrillary tangles, and was not associated with increased phosphorylation of endogenous tau until later life.

Quantitative analysis of immunohistochemistry was undertaken using mAb 7/51 (Fig. 3c, d). In L1 mice aged 3–6 months, the log count of neurons labelled was 14 % of the number labelled with mAb 7/51 in L66 for all regions. At this age, labelling was restricted primarily to entorhinal cortex and hippocampus, with minimal labelling in neocortical regions. In older animals aged 12–18 months, labelling increased in hippocampus and entorhinal cortex, and most notably spread to retrosplenial cortex, subiculum and visual cortex. Thus L1 mice showed the characteristic pattern of progression that is seen as Braak staging in Alzheimer’s disease.

#### Minimal sensorimotor deficits in L1 mice at 3 and 6 months

Unlike L66, L1 mice did not show any gross motor abnormality on cage side observation. When investigated further, only mild differences in gait were observed, being seen as an increase in stride length (Fig. 6e), hindlimb base of support (Fig. 6g), and regularity index (Fig. 6h). Relative paw position (Fig. 6f) did not differ from wild-type mice.

Also in marked contrast to L66, L1 mice showed better performance on the balance beam task than wild-type controls. Independent of beam shape (genotype factor: square Fig. 7c: *F*(1, 52) = 10.3, *p* = 0.004; round Fig. 7d: *F*(1, 52) = 4.5, *p* = 0.04), L1 mice were faster to reach the end of the beam, but all animals found the smaller beams more challenging (beam factor: *F* > 14, *p* < 0.0001). Nevertheless, L1 mice still performed better on the smallest beam than wild-type controls (*t* = 2.46, *p* < 0.05).

We investigated the possibility that a lowering of anxiety might contribute to this behavioural enhancement, but were unable to find any anxiety-related phenotype using the light/dark box. There was no difference in time spent in the dark compartment (Fig. 7e) or the number of entries between light and dark chambers (Fig. 7f: all *p* values >0.2).

Despite faster climbing on beams, L1 mice were impaired in motor coordination and learning on the RotaRod task. When placed on the rotating rod, L1 mice fell off earlier during the first two trials (gene factor: *t* = 4.2, *df* = 54, *p* < 0.0001) than did wild-type mice (Fig. 8g). This suggests a difficulty in movement and step progression in line with the accelerating rod. L1 mice were also slower in acquiring the RotaRod learning task, lagging behind wild-type controls in terms of time on the rotating rod, but nevertheless showing improvement over successive trial days (Fig. 8h; gene factor: *F*(1, 286) = 15.8, *p* = 0.0005). Indeed, the overall gain after 12 trials relative to trial 1 did not differ (Fig. 8i), suggesting equivalent motor learning, albeit at an impaired overall level.

#### Prominent cognitive-spatial deficits in L1 mice at 3 and 6 months

Cognitive phenotyping of L1 mice in the open field water maze was undertaken using two different paradigms. In the first paradigm, we administered a standard reference memory test with constant platform locations over 4 training days (Fig. 9). One wild-type mouse was excluded for continuously poor performance not improving over the course of training. Already at 3 months of age, L1 mice were impaired in acquisition learning as indicated by a longer daily path length to reach the hidden platform (Fig. 9e; gene factor: *F*(1, 68) = 11.8, *p* = 0.003). Both genotypes improved over days (day factor: *F*(4, 68) = 2.8, *p* = 0.03) and reached the same performance level at day 4. There was no evidence of sensory motor deficit, as both groups performed equally in trial 1 (*t* = 0.6, *p* = 0.5). L1 mice always swam faster than controls (Fig. 9f; *F*(1, 63) = 22.5, *p* = 0.0001) but showed normal thigmotaxis (Fig. 9g; *F* = 1). In the probe test, when the target platform was removed, wild-type controls clearly preferred the target over non-target quadrants (Fig. 9h; *t* = 3, *p* = 0.01), but the L1 mice did not. However, this difference was not statistically significant at 3 months, due to high variability (*F* = 2.5, *p* = 0.24; *F* test).

The cognitive deficits became more pronounced at 6 months (Fig. 9i–l). A single wild-type mouse presented with circling behaviour and was excluded from the analysis. L1 mice were consistently impaired and swam longer paths to reach the platform (Fig. 6i; gene factor: *F*(1, 80) = 18, *p* = 0.0004), but were not significantly different in first trial (*t* = 2.1, *p* = 0.08). Both genotypes improved their performance throughout training (day factor: *F*(4, 80) = 9.9, *p* < 0.0001) and differed significantly on day 4 (*t* = 3.9, *p* < 0.001). As already indicated at 3 months, 6-month L1 mice swam faster throughout (Fig. 9j; gene factor: *F*(1, 60) = 26.3, *p* < 0.0001). They became more thigmotaxic than at 3 months (Fig. 9k; gene factor: *F*(1, 60) = 11.8, *p* = 0.003). In the probe test, when the platform was removed, wild-type mice were spatially biased towards the target quadrant over non-target quadrants (*t* = 5.1, *df* = 16, *p* < 0.0001) whereas L1 spent equal times in all quadrants (Fig. 9l). These data demonstrate spatial learning and memory difficulties in L1 mice that begin at 3 months and progress in severity by 6 months.

**Fig. 10:**
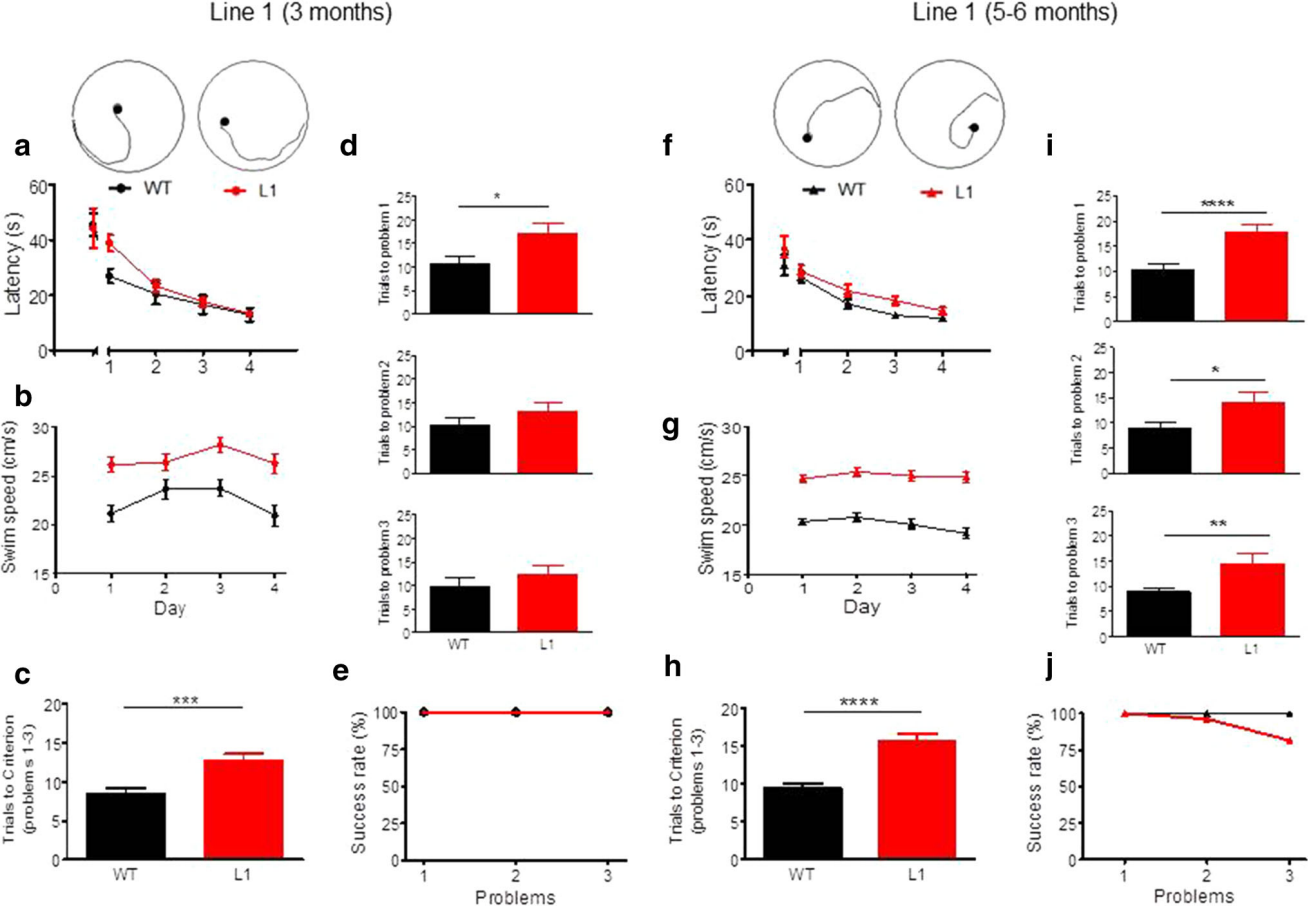
Spatial problem solving task confirms cognitive impairment in L1 tau transgenic mice at 3 months of age (**a**–**e** WT *n* = 10, L1 *n* = 14) and at 5–6 months (**f**–**j** WT *n* = 27; L1 *n* = 27). **a** Latency to reach visible platform during 4 days of learning (4 trials/day). Platform positions were changed in each trial. Note that L1 mice were slow to acquire the task on day 1 (trials 2–4; ***p < 0.001), but not in the initial trial or the following days. **b** L1 mice swim faster than wild-type controls on all days of the cued training. **c** Trials to criterion averaged across all three problems. WT mice need fewer trials to meet criterion than L1 mice. **d** Trials required to meet criterion for problem 1 (*top*), problem 2 (*centre*) and problem 3 (*bottom*). For all problems, WT mice achieve criterion faster than L1 mice, but this was only significant for problem 1 and the impairment was less obvious for problems 2 and 3. **e** Percentage of wild-type and L1 animals that meet criterion for problems 1, 2 and 3 in the pre-set time. At this age, all animals achieved criterion and the 2 week training period was sufficient to solve all problems. **f** Latency to reach visible platform. Note that L1 mice differed from WT on all days apart from trial 1 and day 4, when they reached asymptotic levels of performance. **g** Differences in swim speed recorded during the cued training were apparent for all days with L1 mice swimming significantly faster throughout. **h** Trials to criterion averaged across all three problems in 5- to 6-months-old mice. WT mice acquire criterion much faster than L1 mice. **i** Trials required to meet criterion for problem 1 (*top*), problem 2 (*centre*) and problem 3 (*bottom*). For all problems, WT mice achieve criterion significantly faster than L1 mice. **j** Percentage of wild-type and L1 animals that meet criterion for problems 1, 2 and 3 in the predetermined time. By 5–6 months of age, all wild-type animals solved the three problems, whereas only 82 % of L1 did. Values expressed as group mean + SE. *Asterisks* indicate *p < 0.05, **p < 0.01, ****p < 0.0001

We used a second paradigm to investigate the spatial learning deficit further at 3 months and 5–6 months in separate cohorts of female L1 mice and wild-type controls using a problem solving task in the water maze (Fig. 10). This entailed a visual training over 4 days with platform location shifting between days. Although 3-month-old L1 mice required a longer path to find the platform on day 1 (Fig. 10a; gene factor: *F*(1, 88) = 10, *p* = 0.004) there was no difference for the first and last trial of training. Again, L1 mice swam faster in all trials (Fig. 10b; gene factor: *F*(3, 66) = 17, *p* = 0.0004). Each animal was then required to find the platform in less than 20 s in three consecutive trials. Once the criterion was met, the platform was moved to a different position and training progressed until criterion was met for three problems. L1 mice at 3 months required more trials to achieve criterion when averaged across all problems (Fig. 10c; *t* = 3.4, *df* = 110, *p* = 0.0009). Although L1 mice performed worse in all problems, they improved continuously throughout training, but this only reached significance for problem 1 (Fig. 10d; *t* = 2.2, *df* = 22, *p* = 0.037). However, all animals managed to acquire all problems within the given number of ten sessions (Fig. 10e). At age 6 months, L1 mice required longer paths to learn the visible phase of the task (Fig. 10f; gene factor: *F*(1, 208) = 33.5, *p* < 0.0001) but did not differ at day 4 from wild-type controls. Transgenic mice swam consistently faster (Fig. 10g; gene factor: *F*(1, 156) = 75.2, *p* < 0.0001) and required on average about twice as many trials to attain criterion compared with wild-type mice (Fig. 10h; *t* = 5.4, *df* = 154, *p* < 0.0001). At 6 months, L1 mice were deficient in all problems (Fig. 10i; *t* > 2.4, *p* < 0.05) and only 82 % of L1 transgenics completed the task (Fig. 10j). These data confirm a severe deficit in spatial learning in L1 mice which progresses with age from 3 to 6 months.

## Discussion

We report a comparison of endophenotypes of two transgenic mouse lines overexpressing different tau protein constructs using similar promoters. L66 mimics existing FTDP-17 lines by the inclusion of a P301S point mutation, albeit with an additional G335D mutation which enhances tau aggregation in vitro (data not shown). L1 on the other hand is based on the truncated repeat domain tau fragment which accounts for 95 % of tau protein content of PHFs in AD [28]. Although both models are based on overexpression of tau protein, there are major differences in the resultant neuropathological and behavioural phenotypes. In common with previously reported models based on mutant forms of full-length tau, the phenotype in homozygous L66 mice is characterised by an early onset of sensorimotor deficits, gait anomalies and learning deficits restricted to the motor domain, all of which become more severe as mice age. It culminated in a palsy-like dyskinesia at the age of 7 months resembling Parkinsonism in FTDP-17. Noteworthy is the complete absence of a higher cognitive phenotype in L66 despite the severity of neuropathological changes present. By contrast, L1 mice display deficits in spatial learning resembling the difficulties in forming memories within spatio-temporal contexts in AD [49]. These changes are associated with minimal abnormalities in sensorimotor function. There is therefore a marked dissociation between cognitive and sensorimotor expressions of tau-aggregation pathology that depends solely on the form of tau which is overexpressed in the absence of APP overexpression or β-amyloid pathology.

### Pathological and biochemical differences between L1 and L66 mice suggest different but convergent pathways of molecular pathogenesis

We determined, for several transgenic lines generated with both full-length and truncated constructs, the number of cDNA copies integrated into the host genome after pronuclear injection. Despite having a large copy number and expressing mAb 7/51-reactive truncated tau protein in neurons of L1 mice expressing SStau296–390, the apparent level of protein remained low and we did not observe Bielschowsky- or thioflavin-positive neurons. Indeed, there is a lack of overt fibrillary tau aggregates by conventional histological criteria in L1 mice up to an age of 18 months. We have not undertaken an ultrastructural analysis of these aggregates, but they appear similar histologically to those we have previously reported in AD [50]. Such early pre-tangle aggregates of truncated tau are likewise amorphous in character, and at an ultrastructural level are often associated with mitochondria and ER [51, 52]. Overexpression of a substantially longer truncated construct (tau1–391) likewise reached only the pre-tangle stage of pathology in mice [53]. This suggests that tau protein needs to include the C-terminal domain to achieve the configuration necessary for filament assembly.

A particularly striking feature of the tau-aggregation pathology seen in L1 is its anatomical progression out of the entorhinal cortex and hippocampus at 3–6 months of age into retrosplenial cortex, visual cortex and subiculum at 12–18 months. This progression from archicortex into neocortex is typical of Braak staging in AD. The highly stereotyped neuroanatomical spread of tau pathology is now understood in terms of transmission of tau aggregates at the oligomer stage between neurones [7–12]. We have previously reported that template-directed proteolytic processing of tau protein leading to amplification of repeat domain aggregates is a property inherent to the repeat domain of tau which can be demonstrated in vitro in both cell-free and cell-based models [6, 48]. The present demonstration of the same phenomenon in vivo using a tau construct restricted to the repeat domain further extends the earlier findings. The neuroanatomical spread of pathology in L1 is in marked contrast to the anatomically static nature of the L66 pathology. The only previous report of neuroanatomical spread similar to Braak staging has been in PS19/PDAPP mice [1, 21], with an early transentorhinal stage in animals under 4 months, followed by a limbic stage (4–10 months) and then an isocortical stage (>11 months). The present results show that concomitant expression of mutant APP is not necessary for neuroanatomical spread of this kind, and that this property is inherent to the repeat domain of tau.

In contrast to L1, and despite having only two transgene copies, L66 was characterised by abundant intracellular aggregates of tau recognised by mAb 7/51. The aggregates could be stained both by the Bielschowsky stain and by primulin consistent with progression of aggregation to the filamentous stage [50]. This was confirmed by electron microscopy of aggregates having the same histological properties in spinal cord. By comparison L36 and heterozygous L66 mice showed much weaker immunoreactivity per neuron and had fewer tau-positive neurons overall. Therefore the extent of tangle pathology is dependent on gene dose in mutant full-length tau models. Compared with the counts of tau-positive neurones in L1, those seen in homozygous L66 mice are substantially more numerous. Given the much higher copy number for the transgene in L1 the most likely explanation for this difference is much greater clearance of truncated tau directed to the ER by the signal sequence. The pathology identified by mAb 7/51 in L66 also has a much more widespread regional distribution than L1, including midbrain and spinal cord, as well as cortex and basal forebrain. The highest mean counts in L66 were in hippocampus, entorhinal cortex and retrosplenial cortex. Surprisingly the tau-positive neuron count had already attained a steady state level by 3 months, and decreased substantially with age. This could be due either to tangle-mediated neuronal destruction or age-related adaptation to the transgene leading to enhanced clearance. Whatever the reason, the neuranatomically static nature of the tau pathology in L66 is in marked contrast with the age-dependent neuroanatomical spread observed in L1.

In L66, prominent staining of axons was seen with the phosphorylation dependent mAb AT8. This was not seen with mAb 7/51 which tended to be more restricted to cell bodies and apical dendrites, the typical distribution of PHFs in AD. Although there was a general tendency for regions and cells to be labelled by both antibodies, more detailed analysis showed that there was limited overlap between the two. This was demonstrated by double labelling in L66 mice, where only a subset of neurons with tau aggregates detected with mAb 7/51 were also labelled with mAb AT8. The same phenomenon was demonstrated in aged L1 mice using p-Tau (S-404). Whereas no pS404 immunoreactivity was seen in younger animals, labelling was seen in mice at 15 months in both neocortex and hippocampus. Since the epitope in question is lacking in the transgene, terminating as it does at Ala-390, acquisition of pS404 immunoreactivity implies that both recruitment of endogenous mouse tau and secondary phosphorylation occur only after aggregation has been initiated. One-to-one recruitment of native tau by truncated tau aggregates has been reported previously in rats expressing a longer truncated tau transgene (residues 150–390; [33]).

The implied dissociation between tau aggregation and tau phosphorylation has important theoretical implications, since it is commonly thought that hyperphosphorylation is on the critical path to both aggregation and loss of microtubule-binding function [54]. We have shown previously that, although the quantity of phosphorylated tau in PHF preparations from AD brain is correlated with the total quantity of aggregated tau, phosphorylated tau accounts for less than 5 % of the tau content of the PHF in AD. We have also shown that phosphorylation inhibits tau–tau binding through the repeat domain by a factor of 20- to 50-fold in vitro [55] in line with other reports [5, 28, 56, 57]. As we have argued elsewhere, on the basis of data from human brain tissues, phosphorylation of tau is likely to be a late-stage epiphenomenon in the process of tau aggregation [5, 28]. This is now supported by the L1 transgenic mouse data where tau phosphorylation is seen only after 15 months. In L1, therefore, tau phosphorylation cannot explain the severe cognitive deficits we have documented from 3 months of age.

The prominent axonal staining with mAb AT8 in L66 has been reported previously in other four-repeat tau transgenic mice [58, 59]. A similar pattern of argyrophilic axonal staining has been reported in rats subjected to experimental blast injury [60]. In these rats, it was considered that the pattern of staining (i.e., with a predilection for deep cerebellar white matter) represented a manifestation of the physics of the air blast shock wave. Based on the findings in amino cupric silver-stained sections from L66 mice, it is possible that the pattern of axonal staining may indicate that axons in certain brain regions are inherently more susceptible to degeneration following a variety of stresses. Hyperphosphorylation of tau is known to be a non-specific response to a number of stressors in cell models [61, 62].

**Fig. 11:**
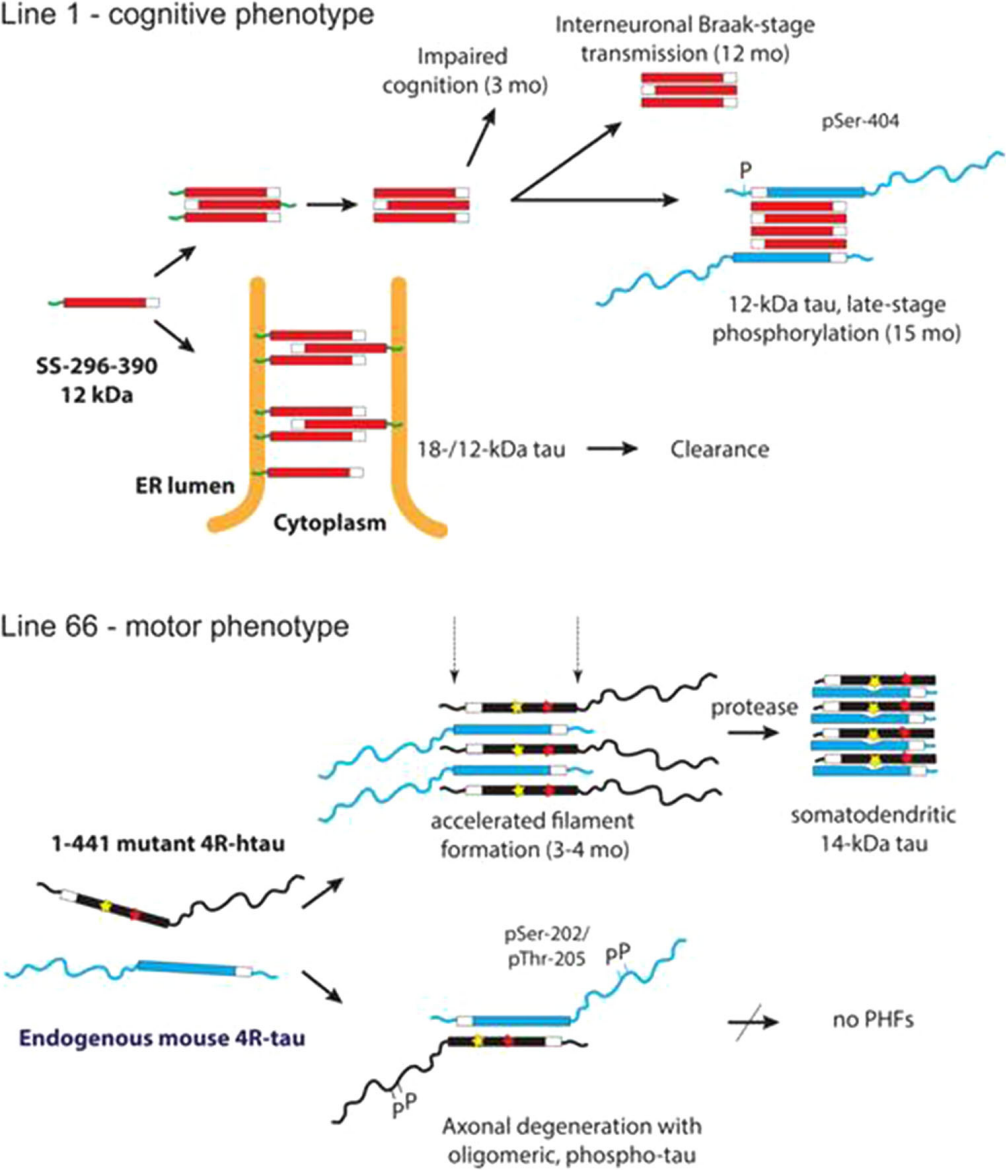
Schematic representation of the molecular pathogenesis for tau in L1 and L66 transgenic mice. In L1 mice, a generally toxic truncated tau species can assemble into aggregates of small oligomers of tau either on its own or via membrane-anchored species targeted to the endoplasmic reticulum membrane when expressed as a fusion with a signal sequence. These oligomers impair cognition already at 3 months and this progresses with age. At later stages, the aggregation pathology can be transmitted in a Braak stage-like fashion to neighbouring neurons which spreads and amplifies the pathology. Phosphorylation of endogenous N-terminal tau that is recruited to the pathological cascade is a late-stage event. A balance between toxicity of truncated tau oligomers and their proteolysis and clearance is required by the mouse for survival. L66 is characterised by an early onset of aggregate and filament formation that is accelerated by the presence of mutations within the PHF-core domain. There are sub-populations of neurons which accumulate filamentous aggregates of tau that consist predominantly of truncated 14-kDa tau oligomers whereas others become phosphorylated, lead to axonal degeneration but do not progress to PHFs. L66 mice are characterised by prominent sensorimotor deficits

The comparison of the L1 and L66 phenotypes at the biochemical and pathological levels demonstrates the existence of two distinct but convergent pathways for the pathological processing of tau protein. These are summarised in Fig. 11. In L1, although pathology is arrested at the oligomer stage, the model nevertheless demonstrates the key features AD, namely prominent cognitive impairment and age-related neuroanatomical spread of pathology of the type formalised as Braak staging. We conclude that oligomers consisting of the truncated repeat domain fragment of the PHF core are the fundamental toxic species required for both phenomena. The low levels of tau pathology, despite high copy number, most likely represents enhanced clearance of the SStau296–390 via the endosomal-lysosomal pathway. This can be contrasted with L66, in which there is early aggressive formation of filamentous neurofibrillary tangles with high levels of pathology in entorhinal cortex and hippocampus occurring in the absence of either higher cognitive deficit or neuroanatomical spread of the Braak staging type. In agreement with others [63, 64], this filamentous/tangle stage of tau protein aggregation is not a critical driver of clinical dementia, although it is linked quantitatively to dementia [18] and eventual tangle-mediated neuronal loss at advanced stages [65]. Hyperphosphorylation of full-length tau is not required for cognitive impairment, interneuronal transmission of pathology, assembly of PHFs or formation of neurofibrillary tangles, but, in a non-aggregating form, appears to be closely linked to disintegrative axonal degeneration. Both pathways involve recruitment of normal tau. Both pathways converge at the point of formation of aggregates in which the proteolytically stable core consisting of a repeat domain fragment is restricted to approximately 3 repeats irrespective of either the transgene or the endogenous tau which is recruited.

### Different learning and sensorimotor impairments in L1 and L66

There was a striking difference in deficits in higher cognitive function between L1 and L66. We employed two different water maze paradigms including a standard open field reference memory task commonly used for tau mice (e.g., [66]) and a problem solving task [46, 67, 68]. The spatial problem solving paradigm is analogous to the delayed-matching-to-place (DMP) task and both have been proposed as behavioural models of working/episodic-like memory in rodents [46, 69, 70]. While the platform was changed daily in the DMP task so that spatial information is useful only for a short period (i.e., one trial), problem solving consisted of repeated acquisition learning of a new platform location to a level meeting a pre-set criterion. Consequently, the spatial information is used for more than a single trial and does not formally fit the classical definition of the “working memory” task [71]. The existence of a strong long-term memory component in the problem solving task makes it more akin to long-term “episodic” memory [72]. Early stages of AD display difficulties in forming new memories of events within specific spatio-temporal contexts and are the earliest and perhaps the most sensitive sign of AD [49]. It is particularly striking that L1 mice already exhibited prominent cognitive deficits by the early age of 3–4 months, and these deficits became more severe at 5–6 months. At this time, tau-aggregation pathology was largely restricted to entorhinal cortex and hippocampus. Conversely, despite the presence of much more extensive pathology in the same regions in L66 homozygous mice at 3 and 6 months, there was no evidence of impairment on the spatial memory task. Several other mouse tau models over-expressing P301S [73], P301L [66, 74] or V337M [75] have also failed to show spatial deficits in the water maze at ages when tangles could be readily detected. On the other hand, truncated tau gene expression in rats was also found to compromise spatial cognition at 4–5 months [33], although the phenotype is confounded at older ages by the emergence of severe motor deficits. The presence of early higher cognitive deficits in both truncated tau models argues that overexpression of a truncated tau fragment containing the repeat domain is an important determinant of cognitive impairment. Indeed, it appears to be more important than high tangle counts in hippocampus and entorhinal cortex, which are substantially higher in L66 than in L1 but without causing higher cognitive deficits.

In addition to the severe deficits in spatial cognitive function in L1 mice, there was also a performance deficit in the RotaRod reminiscent of a global motor impairment. However, their learning curve and overall improvement during training did not differ from controls (Fig. 8). By contrast, in L66 there was no motor impairment (with the exception of elderly, homozygous mice developing palsy-like dyskinesia), but a severe impairment in motor learning. There is therefore an inverse dissociation between the two transgenic mouse lines. Spatial learning in the water maze was impaired for L1 mice, but L66 mice were normal. Conversely, L66 mice were severely impaired in motor learning, but for L1 mice there was no difference in motor learning relative to wild-type animals. These results confirm earlier reports showing that motor dysfunction [25, 76], increased exploration [77] and impairment of motor learning [66, 78, 79] are common phenotypes for FTDP-17 models.

There were likewise marked differences in sensorimotor function in L1 and L66. Six-month-old L1 mice displayed shorter latencies to traverse all square/round beams when compared to age-matched wild-type controls. This finding is also in agreement with the results from young transgenic rats expressing human truncated tau (tau151–391), which were unimpaired in beam walking when 4–6 months old, but progressed to sensorimotor disturbances at the age of 9 months [33]. Similar observations were reported for young tau-P301L transgenic mice vis-a-vis synaptic plasticity and recognition memory [80] and here we report that heterozygous L66 mice carrying the P301S/G335D mutations also exhibited shorter latencies, especially when tested on small beams. By contrast, homozygous L66 mice with and without dyskinesia were severely impaired. It thus appears that the occurrence of sensorimotor phenotypes is dependent both on the dose of gene expression and the type of tau expressed.

Interestingly, the observation of better performance in the balance beam test was coincident with similar changes in gait in L1 and L66 heterozygous mice, with longer stride length and wider hindlimb gait present in both lines. Homozygous L66 mice, on the other hand, show a reduction in these parameters. No comparative data from other tau models are available here, but since the weight or size of L1 and L66 cannot explain these contrasting findings, it is reasonable to suggest that weaker tau expression (be it truncated or full length) may provide some benefits to sensorimotor function. Alternatively, shorter latencies to traverse rods in L1 mice may reflect a hyperactive behavioural profile also seen in heightened swim speed in the water maze. Tau transgenic mice commonly display a hyperactivity phenotype [66, 73, 75, 80] which may provide analogues to symptoms of agitation, disinhibition and hyperactivity seen in AD and in some of the FTLD syndromes [81].

In summary, we report two new tau transgenic mouse models which demonstrate that different molecular pathways underlie motor and cognitive tauopathy phenotypes. The first (L66), based on overexpression of full-length tau with two pathogenic point mutations, is characterised by axonopathy and aggressive filamentous tau-aggregation pathology which is neuroanatomically static and is associated with prominent sensorimotor deficits occurring in the absence of a higher cognitive phenotype. The second (L1), based on overexpression of the truncated repeat domain tau fragment which constitutes the bulk of the PHF-core in AD, shows evidence of neuroanatomical spread and amplification with age that resembles Braak staging in AD and is associated with severe cognitive impairments that progress with age occurring with minimal sensorimotor impairment. In both models, tau aggregation can be dissociated from abnormal phosphorylation. The two models make possible the demonstration of two distinct but nevertheless convergent pathways of tau molecular pathogenesis and show that aberrant processing of APP is not required to explain the differences in the clinical presentations of AD-like and FTLD syndromes.
